# Optimal allocation of STATCOM for multi-objective ORPD problem on thermal wind solar hydro scheduling using driving training based optimization

**DOI:** 10.1038/s41598-025-02636-1

**Published:** 2025-06-04

**Authors:** Tushnik Sarkar, Sabyasachi Gupta, Chandan Paul, Susanta Dutta, Provas Kumar Roy, Anagha Bhattacharya, Ghanshyam G. Tejani, Seyed Jalaleddin Mousavirad

**Affiliations:** 1 Department of Electrical Engineering, Dr. B. C. Roy Engineering College, Durgapur, India; 2 Department of Electrical Engineering, NIT Mizoram, Aizawl, India; 3 Department of Electrical Engineering, Kalyani Government, Kalyani, West Bengal India; 4 Department of Research Analytics, Saveetha Dental College and Hospitals, Saveetha Institute of Medical and Technical Sciences, Saveetha University, Chennai, 600077 India; 5 Department of Industrial Engineering and Management, Yuan Ze University, Taoyuan, 320315 Taiwan; 6 Department of Computer and Electrical Engineering, Mid Sweden University, Sundsvall, Sweden

**Keywords:** Optimal reactive power dispatch (ORPD), Renewable energy sources (RESs), Driving training based optimization (DTBO), Static synchronous compensator (STATCOM), Electrical and electronic engineering, Applied mathematics

## Abstract

On IEEE 30, 57, 118 & 300-bus experimental networks, this work aims to solve the optimal reactive power dispatch (ORPD) problem. Initially, the conventional network is countered, and subsequently, renewable energy sources (RESs) such as wind power (WP), solar photovoltaic (PV) sources, and hydro power (HP) are combined with the traditional network. This study examines both single and multiple type objective functions (OFs). The Objectives include lowering active power loss (APL), lowering aggregated voltage deviation (AVD), lowering the voltage stability index (VSI), lowering reactive power loss and concurrently lowering AVD, APL & VSI. There are five test modules that comprise a total of 30 cases. Cases 5-8 and 13-30 are being conducted using STATCOM in conjunction with the test setup. The Driving Training Based Optimization (DTBO) method has been used to achieve the goals, and its performance has been compared to that of other optimization algorithms that have been reported in recent ORPD studies. Both stable load demand and uncertain changing load demand scenarios are included in the study. Appropriate probability density functions (PDF) are employed to estimate the uncertain WP, PV source, HP, and load demand. Uncertain scenarios with variable load demand, wind speed (WS), solar irradiance (SI), and water flow rate (WFR) are created using Monte Carlo simulations (MCS). Based on a range of studied cases, the experiment results show that the DTBO has a significantly stronger ability to solve ORPD challenges than the optimization methods discovered in the most recent ORPD literature. The usage of STATCOM improves power network performance for the ORPD issue, which is another significant finding. From simulation results it has been observed that for IEEE 30 bus the average power loss (APL) is 4.5086 MW, utilizing STATCOM the APL is reduced by 5.3% MW, with integrating renewable sources the APL is reduced 41%, and for both STATCOM and renewable sources (RESs) system it decreases to 43.6%. Hence, STATCOM and RES help to reduce the power losses using DTBO approach. Furthermore, average voltage deviation (AVD) improved by 97.4 % with incorporating STATCOM-RESs. Voltage stability index (VSI) improved by 26.9% with scheduling STATCOM and renewable sources (RESs). For the multi-objective situation APL & AVD both simultatiously improved to 5.0701(MW) & 0.1221 (*p*.*u*.), respectively, with incorporating STATCOM and RESs using DTBO. Voltage deviation converges at 40 iterations for with STATCOM but for without STATCOM it takes 80 iterations to converge. Similarly for voltage stability index with STATCOM converge 4 iterations earlier rather than without STATCOM system. Again for large scale IEEE 57 bus system The DTBO approach incorporating STATCOM and RESs provided optimal results. So, for IEEE 30, 57, 118 & 300 bus systems DTBO proves its superiority and robustness satisfactorily. From simulation results it has been observed that for IEEE 30 bus the average power loss (APL) is 4.5086 MW, utilizing STATCOM the APL is reduced by 5.3% MW, with integrating renewable sources the APL is reduced 41%, and for both STATCOM and renewable sources (RESs) system it decreases to 43.6%. Hence, STATCOM and RES help to reduce the power losses using DTBO approach. Furthermore, average voltage deviation (AVD) improved by 97.4 % with incorporating STATCOM-RESs. Voltage stability index (VSI) improved by 26.9% with scheduling STATCOM and renewable sources (RESs). For the multi-objective situation APL & AVD both simultatiously improved to 5.0701(MW) & 0.1221 (*p*.*u*.), respectively, with incorporating STATCOM and RESs using DTBO. Voltage deviation converge at 40 iterations for with STATCOM but for without STATCOM it takes 80 iterations to converge. So, for IEEE 30, 57, 118 & 300 bus systems DTBO proof its superiority and robustness satisfactorily.

## Introduction

Optimal reactive power dispatch (ORPD) has an significant role for proper planning & operation of existing power networks. In order to keep the voltages at all system buses within acceptable ranges and to minimize network APL, reactive power needs to be controlled and managed in the system. The majority of system loads are inductive, and since reactive power is used by components like transformers and transmission lines, it cannot be avoided in the system. As reactive power flow results in APL, ORPD sets the minimizing of system APL as its major goal. A typical network will alter the passive tool settings like transformers and shunt VAR compensators to get the desired result. Power researchers are therefore making constant efforts to reduce predetermined OFs without violating a range of system restraints in order to address ORPD difficulties^1–3^..

To achieve the goals within the allowed system limitations (both equality and inequality), the most advantageous adjustment of specific control variables are the generating buses’ voltages, the tap settings of the transformer, the distribution of the VAR shunt compensator etc.^4,5^.

Presently integrating RESs with the conventional power grid is becoming gradually popular for the reasons of sustainability. However, the character of RESs is not deterministic rather stochastic. As result, introduction of RESs with traditional configurations enhances system complexity and makes achieving the ORPD solution is a more difficult task^6^. The modern advancement of power electronic technologies enhances the utilization of flexible AC transmission system (FACTS) devices (like flexible, stable & dependable VAR compensators) to effectively address the ORPD issues^7^.

The reduction of AVD, the diminution of APL etc are very often chosen as the OFs in the area of ORPD studies. Conventional optimization techniques, such as dynamic programming, linear programming, and others, have been discussed in the literature. However they are unable to solve non-differentiable functions. These methods took more iterations to generate results, therefore they were more time consuming. More often they were producing a local optimal solution instead of global solutions. Moreover, these traditional approaches of optimizations were inefficient to handle complex, non-linear optimization problem. Thus, more advanced optimization techniques have been incessantly created, and being applied on different power system applications, like ORPD problem which addresses the shortcomings of earlier approaches in solving ORPD problem^8,9^. Presently, development and utilization of meta heuristic methods have demonstrated successful outcomes in accomplishing ORPD difficulties^10^.

Table 1 is represented here to show a brief contemporary situations of ORPD research where different optimization algorithms has been applied to achieve several single OFs like declining APL, reduction in AVD, VSI enhancement, fuel cost reduction, reduction in emission, operational cost minimization, and uniting more OFs together as a multi-OFs^11^.

Laouafi^12^ proposed improved grey wolf optimizer (IGWO) to get the solution of the problem of optimal reactive power dispatch (ORPD). The effectiveness of the method was tested on the IEEE 30 bus test system with and without solar and wind energy as renewable energy resources (RESs). Megantoro *et* *al*^13^. used meta-heuristic algorithm to sort out the ORPD problem. Here, Wind and solar power are used as RESs. This technique was tested on IEEE 57 Bus system for identifying the robustness. In the aforesaid paper, objective functions of power loss, minimize voltage deviation, and improvement the voltage stability index (VSI) were minimize. Das *et* *al*^14^. used the rock hyraxes swarm optimization (RHSO) algorithm to find the solutions of ORPD problem. Paul *et* *al*^15^. implemented chaotic-oppositional (CO) based DTBO approach in CHPED based OPF problem by considering wind-solar-EV to minimize the generation cost and emission and to improve the voltage deviation. This proposed technique was tested on the IEEE 33 and IEEE 141 bus systems with and without PV-Wind power. Tu *et* *al*^16^. suggested an improved multi-objective equilibrium optimizer (IMOEO) for fixing the OARPD issue with renewable sources. The procedure is used to an modified IEEE-33 distribution network to check its performance. Hasanien *et* *al*^17^. proposed hybrid particle swarm Optimization/sea horse optimization (PSOSHO) algorithm to handle ORPD for electric vehicle integrated system. It was tested on IEEE 30-bus and IEEE 57-bus networks to verify its efficacy. Paul *et* *al*^18^. applied COWOA optimization to analyze hydro-thermal scheduling problem integrated with wind and solar for optimal solution of cost and emission. Nagrajan^19^ *et* *al*. focused on the enhanced wombat optimization algorithm (EWOA) for solving the optimal power flow (OPF), taking into account the RES-solar photovoltaic (PV) system, Wind energy (WE), Electric vehicles (EVs). The potential of this optimization technique was checked by applying it over IEEE 30-, IEEE 57-, & IEEE 118-bus networks. Ahmed *et* *al*^20^. applied gradient jellyfish search optimizer (GJSO) to accomplish the ORPD issue in electric networks. It was conducted on typical IEEE-30 & IEEE-57 bus systems to measure the effectiveness of the GJSO methodology. Chandra *et* *al*^21^. suggested an approach to analysis the voltage stability in the grid for ORPD problem. Elkholoy *et* *al*^22^.proposed an approach to improve the power quality in the distribution network (IEEE 13 bus) for unbalance load with utilizing different FACTS devices. Chandra *et* *al*^23^.applied competitive swarm technique integrated with oppositional-based learning to find the optimal location of the solar charging station for EV in the radial distribution. Split bregman approach applied by Rong *et* *al*^24^. for ORPD in induction generator in a wind power plant.

It is clear from Table 1 that as of 2023, there are still researchers striving to enhance ORPD solutions, hence ORPD research has not arrived its zenith yet. When the Table 1 which presents an overview of the nearly five years of ORPD study, is quantitatively evaluated, it is discovered that, out of 37 investigations, the IEEE 30-bus network was selected in 30 times, giving it a higher preference than other IEEE bus systems, as seen in Table 1. When the various OFs that were considered in those studies are totaled, it is found that every study has chosen lowering APL as one of the OFs, and 26 investigations have also considered decreasing AVD. This suggests that declining APL and AVD are the most common OFs in ORPD. The current work, which uses the IEEE 30-bus test system to achieve the bare minimum APL and AVD, is motivated by these quantitative studies.

**Table 1:** Different ORPD research from literature.

References	Year	Used algorithm for resolving ORPD	Test system								Objective function					
			Benchmark Functions	IEEE 14-Bus System	IEEE 30-Bus System	IEEE 39-Bus System	IEEE 57-Bus System	IEEE 114-Bus System	IEEE 118-Bus System	IEEE 300-Bus System	Reducing APL	AVD diminution	VSI enrichment	Cost cutting	Lessening Emission	Effects of Uncertainty
^25^	2019	Success history based adaptive differential evolution			$\checkmark$		$\checkmark$				$\checkmark$	$\checkmark$				
^26^	2019	Modified sine cosine algorithm			$\checkmark$						$\checkmark$	$\checkmark$	$\checkmark$			
^27^	2019	Improved antlion optimization algorithm			$\checkmark$		$\checkmark$		$\checkmark$		$\checkmark$	$\checkmark$	$\checkmark$			
^28^	2019	Hybrid artificial physics-particle swarm optimization			$\checkmark$		$\checkmark$		$\checkmark$		$\checkmark$	$\checkmark$	$\checkmark$			
^29^	2019	Enhanced grey wolf optimizer			$\checkmark$						$\checkmark$	$\checkmark$				$\checkmark$
^30^	2019	Modified salp swarm algorithm			$\checkmark$			$\checkmark$			$\checkmark$	$\checkmark$				
^9^	2020	Chaotic Bat algorithm		$\checkmark$		$\checkmark$	$\checkmark$		$\checkmark$	$\checkmark$	$\checkmark$	$\checkmark$	$\checkmark$			
^2^	2020	Fractional-order Darwinian particle swarm optimization			$\checkmark$		$\checkmark$				$\checkmark$	$\checkmark$				
^31^	2020	Improved social spider optimization algorithm	$\checkmark$		$\checkmark$				$\checkmark$		$\checkmark$	$\checkmark$	$\checkmark$			
^32^	2020	Marine predators’ algorithm			$\checkmark$						$\checkmark$	$\checkmark$				$\checkmark$
^33^	2020	Water wave optimization			$\checkmark$						$\checkmark$					
^34^	2020	Artificial bee colony algorithm			$\checkmark$		$\checkmark$				$\checkmark$	$\checkmark$	$\checkmark$			
^35^	2020	Jaya algorithm		$\checkmark$	$\checkmark$						$\checkmark$					
^36^	2020	Improved Lightning Attachment Procedure Optimization (LAPO)			$\checkmark$						$\checkmark$					
^37^	2021	Merchant Optimization Algorithm (MOA)		$\checkmark$	$\checkmark$			$\checkmark$			$\checkmark$					
^38^	2021	Hybrid grey wolf optimization and particle swarm optimization		$\checkmark$	$\checkmark$				$\checkmark$		$\checkmark$	$\checkmark$				
^39^	2021	Improved slime mould algorithm	$\checkmark$				$\checkmark$		$\checkmark$	$\checkmark$	$\checkmark$					
^40^	2021	Modified pathfinder algorithm					$\checkmark$		$\checkmark$		$\checkmark$					
^41^	2021	Improved Heap-based optimizer			$\checkmark$		$\checkmark$		$\checkmark$		$\checkmark$	$\checkmark$	$\checkmark$			
^42^	2021	Artificial ecosystem optimization			$\checkmark$				$\checkmark$	$\checkmark$	$\checkmark$	$\checkmark$	$\checkmark$			
^43^	2021	Sun flower optimization			$\checkmark$						$\checkmark$		$\checkmark$			
^44^	2021	Hybridization of genetic particle swarm optimization algorithm with symbiotic organisms search algorithm			$\checkmark$						$\checkmark$	$\checkmark$	$\checkmark$			
^45^	2021	Dragonfly optimization algorithm			$\checkmark$						$\checkmark$					
^46^	2022	Chaotic turbulent flow of water-based optimization			$\checkmark$		$\checkmark$				$\checkmark$	$\checkmark$				
^47^	2022	Continuous Ant Colony-based Differential Evolution			$\checkmark$		$\checkmark$				$\checkmark$		$\checkmark$			
^48^	2022	Teaching and learning based optimization		$\checkmark$	$\checkmark$		$\checkmark$				$\checkmark$			$\checkmark$		
^49^	2022	Artificial hummingbird algorithm		$\checkmark$		$\checkmark$					$\checkmark$	$\checkmark$				$\checkmark$
^50^	2022	Coronavirus Herd Immunity Optimizer			$\checkmark$		$\checkmark$		$\checkmark$		$\checkmark$		$\checkmark$			
^51^	2022	Dynamic exploitation Gaussian bare-bones bat algorithm		$\checkmark$			$\checkmark$		$\checkmark$		$\checkmark$	$\checkmark$	$\checkmark$			
^52^	2022	Modified jellyfish optimizer			$\checkmark$						$\checkmark$	$\checkmark$	$\checkmark$			$\checkmark$
^53^	2022	Improved aquila optimization		$\checkmark$	$\checkmark$		$\checkmark$		$\checkmark$		$\checkmark$	$\checkmark$	$\checkmark$			
^54^	2022	Hybrid fuzzy evolutionary algorithm		$\checkmark$							$\checkmark$					$\checkmark$
^6^	2023	Modified artificial hummingbird algorithm			$\checkmark$						$\checkmark$	$\checkmark$	$\checkmark$			$\checkmark$
^55^	2023	Augmented social network search			$\checkmark$		$\checkmark$		$\checkmark$		$\checkmark$	$\checkmark$	$\checkmark$			
^3^	2023	African vultures optimization			$\checkmark$		$\checkmark$		$\checkmark$		$\checkmark$	$\checkmark$		$\checkmark$		
^56^	2023	Improved Barnacles Mating optimizer					$\checkmark$				$\checkmark$					
^57^	2023	Enhanced Jaya and Artificial Ecosystem-based optimization			$\checkmark$		$\checkmark$				$\checkmark$	$\checkmark$				

Here, based on the selection of OFs, kind of load demand and test settings we have developed 30 distinct cases which are covered in five test modules. Trials are being conducted in module one under fixed load with out considering RESs. However, only half of the cases in this module use STATCOM; as FACT device. In both modules one and two, the same strategy has been applied with regard to the use of STATCOM. The second module, in contrast to the first, adds RESs with test setup and runs tests under various load scenarios. Test module three to five are also conducted by considering RESs & STATCOM tools. In these cases, the RESs and changeable load requirements are being modeled using best matched PDFs to capture their uncertainties. Furthermore, utilizing MCS and BRA, 25 plausible situations are generated, over which the testing in the second module are conducted. The goal in creating the scenarios is to replicate the events that take place in actual power networks as accurately as possible. In this study, DTBO algorithm^58^ has been proposed to resolve the ORPD issue. Few graceful members from DTBO population are chosen as driving instructors, while the remaining members are categorized as trainee drivers.

## Model: STATCOM and RESs

### Modeling of STATCOM

**Fig. 1:**
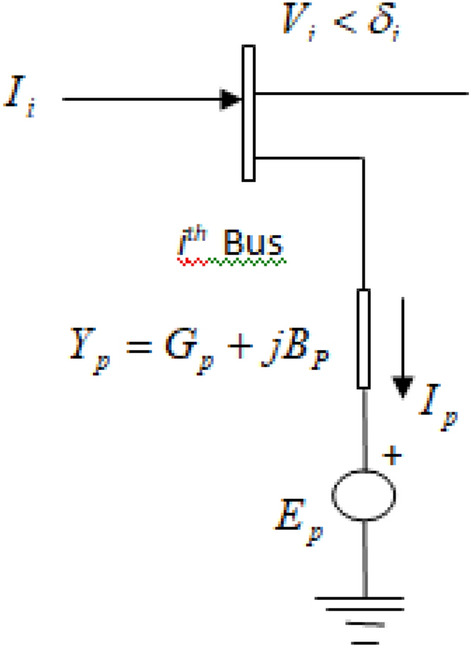
Schematic static model of STATCOM.

To control the power flow, the static synchronous compensator (STATCOM)^59^ device is considered in this experiment. The following is an explanation of this FACTS device’s static model. The main goal of STATCOM is reactive power compensation, which is achieved by varying the power network’s reactive power and voltage magnitude. The components of this device are a transformer, a voltage source converter (VSC), and a capacitor. STATCOM is used parallel with the power system network. A controllable voltage source (${E}_{p}$) in series with an impedance will be used to model the STATCOM. The STATCOM circuit model is shown in Fig. 1, attached to the power system’s ${i}^{th}$ bus.

STATCOM takes in the right extent of reactive electricity through the grid to maintain voltage stability over the power system loads under acceptable limits. The injected active & reactive power flow equations of the ${i}^{th}$ bus are shown below:

1 $$\begin{aligned} & \begin{array}{cc} P_i& =G_p\left| V_i \right| ^{2}-\left| V_i\right| \left| E_p\right| \left| Y_p \right| \cos (\delta _i-\delta _p-\theta _p) \\ & +\sum \limits _{j=1}^{N}\left| V_i\right| \left| V_j \right| \left| Y_{ij}\right| \cos (\delta _i-\delta _j-\theta _{ij}) \\ \end{array} \end{aligned}$$

2 $$\begin{aligned} & \begin{array}{ll} {{Q}_{i}}& =-{{B}_{p}}{{\left| {{V}_{i}} \right| }^{2}}-\left| {{V}_{i}} \right| \left| {{E}_{p}} \right| \left| {{Y}_{p}} \right| \sin ({{\delta }_{i}}-{{\delta }_{p}}-{{\theta }_{p}}) \\ & +\sum \limits _{j=1}^{N}{\left| {{V}_{i}} \right| }\left| {{V}_{j}} \right| \left| {{Y}_{ij}} \right| \sin ({{\delta }_{i}}-{{\delta }_{j}}-{{\theta }_{ij}}) \\ \end{array} \end{aligned}$$

STATCOM brings in two state variables ($\left| {{E}_{p}} \right|$ and ${{\delta }_{p}}$) into the transmission network. In a steady state, it is confirmed that the power used by the source should be zero and represented as

3 $$\begin{aligned} {\begin{matrix} & {{P}_{Ep}}=\operatorname {Re}al[{{E}_{p}}I_{p}^{*}] \\ & \quad \ \,=-{{G}_{p}}{{\left| {{E}_{p}} \right| }^{2}}+\left| {{E}_{p}} \right| \left| {{V}_{i}} \right| \left| {{Y}_{p}} \right| \cos ({{\delta }_{i}}-{{\delta }_{p}}+{{\theta }_{p}}) \\ & \quad \ =0 \\ \end{matrix}} \end{aligned}$$

where ${{V}_{i}}$ is the magnitudes of the voltage at the ${{i}^{th}}$ bus; ${{Y}_{p}}$ is the parallel component’s admittance; ${{B}_{p}}$ and ${{G}_{p}}$ are the susceptance and conductance of STATCOM ‘s parallel components, respectively; ${{\theta }_{ij}}$ is the transmission line’s (placed within ${{i}^{th}}$ and ${{j}^{th}}$ bus) angle of admittance; $\theta _p$ is the angle of STATCOM’s voltage source; $E_p$ is of STATCOM’s voltage sources.

### WP model

Two parameters namely, scale ($\iota$) & shape ($\varkappa$) parameter, provide a good illustration of the WS variation (v m/s)^60,61^ using the Weibull PDF as:

4 $$\begin{aligned} f\left( v \right) =\left( \frac{\varkappa }{\iota } \right) \times {{\left( \frac{v}{\iota } \right) }^{\varkappa -1}}\times \left( {{e}^{-\left( \frac{v}{\iota } \right) }}^{\varkappa } \right) \quad \quad 0<v<\infty \end{aligned}$$

According to the cut-in pace ${{v}_{in}}$, rated pace ${{v}_{r}}$, cut-out pace ${{v}_{out}}$, and output ratting of the wind turbine (WT) ${{P}_{wr}}$, the output power from a WT is given as follows:

5 $$\begin{aligned} {{P}_{w}}(v)=\left\{ \begin{array}{cc}0\\ {{P}_{wr}}\left( \frac{v-{{v}_{in}}}{{{v}_{r}}-{{v}_{in}}} \right) \quad \quad \\ {{P}_{wr}} \\ \end{array}\begin{array}{cc} for\,{{v}_{in}}>v\ \& \ v>{{v}_{out}} \\ for\ {{v}_{in}}\le v\le {{v}_{r}} \\ for\ {{v}_{r}}<v\le {{v}_{out}} \\ \end{array} \right. \end{aligned}$$

Now, the likelihood of WP in various WS zones may be explained by:

6 $$\begin{aligned} & f{{\left( {{P}_{w}} \right) }_{\left| {{P}_{w}}=0 \right. }}=1-\exp \left[ -{{\left( \frac{{{v}_{in}}}{\iota } \right) }^{\varkappa }} \right] +\exp \left[ -{{\left( \frac{{{v}_{out}}}{\iota } \right) }^{ \varkappa }} \right] \end{aligned}$$

7 $$\begin{aligned} & f{{\left( {{P}_{w}} \right) }_{\left| {{P}_{w}}={{P}_{wr}} \right. }}=\exp \left[ -{{\left( \frac{{{v}_{r}}}{\iota } \right) }^{\varkappa }} \right] -\exp \left[ -{{\left( \frac{{{v}_{out}}}{\iota } \right) }^{\varkappa }} \right] \end{aligned}$$

8 $$\begin{aligned} & {\begin{matrix} & f{{\left( {{P}_{w}} \right) }_{\left| 0<{{P}_{w}}<{{P}_{wr}} \right. }}=\left[ \frac{\varkappa \times \left( {{v}_{r}}-{{v}_{in}} \right) }{{{\iota }^{\varkappa }}\times {{P}_{wr}}} \right] \times {{\left[ {{v}_{in}}+\left( \frac{{{P}_{w}}}{{{P}_{wr}}} \right) \left( {{v}_{r}}-{{v}_{in}} \right) \right] }^{\varkappa -1}} \\ & \quad \quad \quad \quad \quad \quad \times \exp \left[ -{{\left( \frac{{{v}_{in}}+\left( \frac{{{P}_{w}}}{{{P}_{wr}}} \right) \times \left( {{v}_{r}}-{{v}_{in}} \right) }{\iota } \right) }^{\varkappa }} \right] \\ \end{matrix}} \end{aligned}$$

In this study, $\xi =10$, $\kappa =2$ & ${P}_{wr}$=80MW have been considered.

### PV model

The function of PV unit is to transform the solar energy into electrical energy. The amount of SI and other environmental factors can affect power output. Since the lognormal PDF L(I) is very much closed with respect to the probability distribution of SI (I : denotes SI)^60,62^, it is frequently used to estimate SI and is expressed as:

9 $$\begin{aligned} L\left( I \right) =\frac{1}{I\lambda \sqrt{2\pi }}\exp \left( \frac{-{{\left( \ln I-\varepsilon \right) }^{2}}}{2{{\lambda }^{2}}} \right) \quad ,\ \ I>0 \end{aligned}$$

$\varepsilon$ & $\lambda$, respectively, represent the mean and standard deviation of the *I* distribution. $\varepsilon =6$ & $\lambda =0.6$ are being chosen here.

The formula for the relationship between SI and the electrical output power of a PV unit is depicted as:

10 $$\begin{aligned} P\left( I \right) =\left\{ \begin{array}{cc} {{P}_{nm}}\frac{{{I}^{2}}}{{{I}_{st}}{{I}_{c}}},\quad for\ 0<I<{{I}_{c}} \\ \\ {{P}_{nm}}\frac{I}{{{I}_{st}}},\quad for\,I\ge {{I}_{c}} \\ \end{array} \right. \end{aligned}$$

The output power nominal of a PV unit, SI standard, & point of critical irradiance are denoted by ${{P}_{nm}}$, ${{I}_{st}}$ and ${{I}_{c}}$ respectively.

### HP model

The behaviour of the fluctuations of WFR is usually modeled using Gumbel PDF^6^, which is expressed as follows:

11 $$\begin{aligned} f\left( {{Q}_{h}} \right) =\left( \frac{1}{\gamma } \right) \times {{e}^{\left( \frac{{{Q}_{h}}-\tau }{\gamma } \right) }}\times {{e}^{-{{e}^{\left( \frac{{{Q}_{h}}-\tau }{\gamma } \right) }}}} \end{aligned}$$

where, with values of 15 and 1.2, respectively, $\tau$ & $\gamma$ denote the location and scale factors of the WFR under consideration. The WFR is $Q_{h}$. The following formula is used to determine the power from the HP-unit.

12 $$\begin{aligned} {{P}_{h}}\left( {{Q}_{h}} \right) =0.85\times \sigma \times \delta \times {{Q}_{h}}\times {{H}_{h}} \end{aligned}$$

The water density is denoted by $\sigma$ which is approximately $1000 kg/m^2$. The gravitational acceleration is represented by $\delta$. 0.85 is the hydro turbine’s efficiency. $H_h$ stands for the water’s head across the turbine.

## Formulating problem

### Objective function

Formulation of single objectives describes^63^ the reduction of APL, AVD, VSI,Reactive power loss & STATCOM installation cost. Below are the explanations of the previously mentioned objectives:

#### APL

Within transmission lines, inherent resistance results in APL. A representation of APL that must be minimized is as follows:

13 $$\begin{aligned} Min{{\mathcal {F}}_{1}}=\sum \limits _{n=1}^{{{N}_{L}}}{{{G}_{n\left( pq\right) }}\left( V_{p}^{2}+V_{q}^{2}-2{{V}_{p}}{{V}_{q}}\cos {{\varphi }_{pq}} \right) } \end{aligned}$$

The ${{n}^{th}}$ line’s transfer conductance, which connects between buses *p* and *q*, is ${{G}_{n(pq)}}$. There are ${{N}_{L}}$ total transmission lines. Between buses *p* and *q*, there is a voltage angle ${{\phi }_{pq}}$

#### AVD

AVD over the load buses should be reserved to a smallest to maintain a decent voltage profile, and it is determined by:

14 $$\begin{aligned} Min{{\mathcal {F}}_{2}}=\sum \limits _{l=1}^{{{N}_{B}}}{\left| {{V}_{l}}-1 \right| } \end{aligned}$$

${{V}_{l}}$ : Voltage at load bus *l*. No. of load buses is ${{N}_{B}}$

#### VSI

The third goal function is to improve voltage stability. Voltage variations result in voltage instability, which can harm power networks or even induce voltage collapse, either suddenly or gradually. VSI needs to be enhanced in order to keep the voltage from dropping. The VSI is presented as per following equations:

15 $$\begin{aligned} & {{F}_{3}}=\min \left( {{L}_{\max }} \right) =\min \left( \max \left( {{L}_{j}} \right) \right) \ \forall \ j=1,2....,{{N}_{b}} \end{aligned}$$

16 $$\begin{aligned} & {{L}_{j}}=\left| 1-\sum \limits _{i=1}^{{{N}_{G}}}{{{F}_{ji}}\frac{{{V}_{i}}}{{{V}_{j}}}} \right| \,\forall \,j=1,2......{{N}_{L}} \end{aligned}$$

Here, $L_j$ represents stability index of $j^{th}$ bus; ${{F}_{ji}}=-{{\left[ {{Y}_{1}} \right] }^{-1}}\left[ {{Y}_{2}} \right]$; $Y_1$ & $Y_2$ are network’s $Y_{BUS}$ sub-matrices.

#### Multi-objective

Multi-objective function^25^ has been formed by taking the linear combination of APL and AVD as:

17 $$\begin{aligned} Min{{\mathcal {F}}_{4}}=APL + \lambda .(AVD) \end{aligned}$$

where $\lambda (=10$), is known as weight factor.

18 $$\begin{aligned} MinF=APL+{{\lambda }_{1}}(AVD)+{{\lambda }_{2}}(VSI) \end{aligned}$$

where $\lambda _1(=10$) and $\lambda _2(=10$) are known as weight factors.

#### Reactive power loss (RPL)

19 $$\begin{aligned} Min{{F}_{s}}=\sum \limits _{n=1}^{{{N}_{L}}}{{{B}_{npq}}}(V_{p}^{2}+V_{q}^{2}-2{{V}_{p}}{{V}_{q}}\operatorname {Sin}{{\phi }_{pq}}) \end{aligned}$$

The ${{n}^{th}}$ line’s transfer susceptance, which is connected between buses *p* and *q*, is ${{B}_{n(pq)}}$. There are ${{N}_{L}}$ number of transmission lines. Between buses *p* and *q*, there is a voltage angle ${{\phi }_{pq}}$.

#### STATCOM installation cost in ($/hr)

The installation cost of STATCOM^64^ is expressed in terms of operating range of the STATCOM in MVA, number of FACTS and capital recovery factors and is given by:

20 $$\begin{aligned} & {{C}_{STATCOM}}=\sum \limits _{j=1}^{D}{({{F}_{STATCOMj}}\times {{S}_{j}}}\times 1000\times \beta )/8760 \end{aligned}$$

21 $$\begin{aligned} & {{F}_{STATCOMj}}=0.0003S_{j}^{2}-0.2691{{S}_{j}}+188.22 \end{aligned}$$

22 $$\begin{aligned} & \beta =\frac{r{{(1+r)}^{n}}}{{{(1+r)}^{n}}-1} \end{aligned}$$

where ${S}_{j}$: Rating of the ${(j)}^{th}$ STATCOM in MVAr; r:interest rate=0.05; n: capital recovery plan for 10 years; $\beta$=0.1295;

### Constraints

The following limitations are applied to the ORPD with STATCOM devices:

#### Equality constraints

Constraint (23) provides a power flow equation which is shown below:

23 $$\begin{aligned} \left\{ \begin{array}{cc} \sum \limits _{c=1}^{{{N}_{s}}}{\left( {{P}_{Gc}}-{{P}_{Lc}} \right) }=\sum \limits _{c=1}^{{{N}_{s}}}{\sum \limits _{d=1}^{{{N}_{s}}}{{{V}_{c}} }{{V}_{d}}}\left( {{g}_{cd}}\cos {{\varphi }_{cd}}-{{h}_{cd}}\sin {{\varphi }_{cd}} \right) \\ \sum \limits _{c=1}^{N _{s}}{\left( {{Q}_{Gc}}-{{Q}_{Lc}} \right) {=-\sum \limits _{c=1}^{{{N}_{s}}}{\sum \limits _{d=1}^{{{N}_{s}}}{{{V}_{c}} }{{V}_{d}}}\left( {{g}_{cd}}\sin {{\varphi }_{cd}}-{{h}_{cd}}\cos {{\varphi }_{cd}} \right) }} \\ \end{array} \right. \end{aligned}$$

Where ${{P}_{Lc}}$, ${{Q}_{Lc}}$: real and reactive load of ${{c}^{th}}$ node (*i*.*e*. *bus*); ${{P}_{Gc}}$, ${{Q}_{Gc}}$: real and reactive generation of ${{c}^{th}}$ node; ${{g}_{cd}}$, ${{h}_{cd}}$ are conductance and susceptance of the $c-d$ branch; ${{\varphi }_{cd}}$ is the admittance angle of the transmission line between $c-d$ nodes.

#### Inequality constraints

(i) Generator constraints:
  24 $$\begin{aligned} \left\{ \begin{array}{cc} V_{Gb}^{\min }\le {{V}_{Gb}}\le V_{Gb}^{\max } \\ \begin{array}{cc} P_{Gb}^{\min }\le {{P}_{Gb}}\le P_{Gb}^{\max } \\ \end{array} \\ Q_{Gb}^{\min }\le {{Q}_{Gb}}\le Q_{Gb}^{\max } \\ \end{array} \right. \begin{array}{ccc} & b\in {{N}_{P}} & \\ \end{array} \end{aligned}$$
(ii) Load bus constraints:
  25 $$\begin{aligned} V_{Lb}^{\min }\le {{V}_{Lb}}\le V_{Lb}^{\max }\begin{array}{cc} & b\in {{N}_{BL}} \\ \end{array} \end{aligned}$$
(iii) Transmission line constraints:
  26 $$\begin{aligned} {{S}_{L}}_{b}\le S_{Lb}^{\max }\begin{array}{cc} & b\in {{N}_{LT}} \\ \end{array} \end{aligned}$$
(iv) Transformer tap constraints:
  27 $$\begin{aligned} T_{b}^{\min }\le {{T}_{b}}\le T_{b}^{\max }\begin{array}{cc} & b\in {{N}_{T}} \\ \end{array} \end{aligned}$$
(v) Shunt compensator constraints:
  28 $$\begin{aligned} Q_{Cb}^{\min }\le {{Q}_{Cb}}\le Q_{Cb}^{\max }\begin{array}{cc} & b\in {{N}_{sc}} \\ \end{array} \end{aligned}$$
(vi) STATCOM voltage and phase angle constraints are respectively depicted in (29) and (30):
  29 $$\begin{aligned} & E_{Sb}^{\min }\le {{E}_{Sb}}\le E_{Sb}^{\max }\begin{array}{cc} & b\in \\ \end{array}{{N}_{STATCOM}} \end{aligned}$$
  30 $$\begin{aligned} & \delta _{Sb}^{\min }\le {{\delta }_{Sb}}\le \delta _{Sb}^{\max }\begin{array}{cc} & b\in \\ \end{array}{{N}_{STATCOM}} \end{aligned}$$

Here $V_{Gb}^{min },V_{Gb}^{max }$ indicate voltage operating range; $P_{Gb}^{min }, ~P_{Gb}^{max }$ represent real power generation operating range; $Q_{Gb}^{min }, Q_{Gb}^{max }$ depict reactive power generation operating range; $V_{Lb}^{min}, V_{Lb}^{max }$ indicate load voltage range; ${{S}_{Lb}}^{min }, S_{Lb}^{max }$ power flow limits of transmission line; $T_{b}^{min }, T_{b}^{max }$ shows tap setting limits; $Q_{Cb}^{min }, Q_{Cb}^{max }$ represent VAr compensation range; $E_{sb}^{\max },~ E_{sb}^{\min }$ indicate voltage range of the STATCOM; $\delta _{sb}^{\max },\delta _{sb}^{\min }$ are phase angle range of STATCOM; ${{N}_{P}}$ depicts generating buses; ${{N}_{BL}}$ represents load buses; ${{N}_{LT}}$ represents transmission line; ${{N}_{T}}$ is the number of regulating transformers; ${{N}_{sc}}$ is the number of shunt compensators and ${{N}_{STATCOM}}$ is count of STATCOM.

## Algorithm for optimization

### Driving training based optimization(DTBO)

DTBO approach is based on driving behaviors and training models. The idea of optimizing driving training-based is to improve driving efficiency, safety, and experience. Driving performance is intended to be optimized through the use of data, driving habits, and continuous input. This involves gathering information on a driver’s driving behaviors, including acceleration, braking, cornering, speed, and fuel consumption. Drivers can receive individual training programs that focus on their particular areas for improvement using the data collected. Reducing pollutants, fuel consumption, and expenses can all be achieved by optimizing driving behavior. This driving behavior helps to provide an optimal solution in optimizing technique with dynamic adaptability.

Dehghani *et* *al*. introduced DTBO at first^58^. DTBO is a population-based meta-heuristic technique. The DTBO program emulates the manner in which a driving instructor instructs trainees in a driving school. The mathematical framework of DTBO contains three phases: (1) training by the driving instructor, (2) patterning of students from instructor skills, and (3) practice. The ability of novice drivers to learn and master the skill of driving depends on their level of intelligence. A seasoned driver can learn from a variety of instructors in driving school. Driving skills are developed by new drivers through practicing on their own and by according to their instructor’s instructions. The foundation of the mathematical modeling of DTBO is these learner-teacher interactions and self-practice for improving driving skills. The following represents the DTBO population matrix, where each row member is one of the possible solutions to the given problem:

31 $$\begin{aligned} Z={{\left[ {\left\{ \begin{array}{ll} & {{Z}_{1}} \\ & . \\ & . \\ & {{Z}_{p}} \\ & . \\ & . \\ & {{Z}_{N}} \\ \end{array}\right. } \right] }_{N\times m}}={{\left[ {\left\{ \begin{array}{ll} & {{z}_{11}}\quad .\quad .\quad {{z}_{1q}}\quad .\quad {{z}_{1m}} \\ & .\quad \quad .\quad .\quad .\quad \quad .\quad \ . \\ & .\quad \quad .\quad .\quad .\quad \quad .\quad \ . \\ & {{z}_{p1}}\quad .\quad .\quad {{z}_{pq}}\quad .\quad {{z}_{pm}}\ \\ & .\quad \quad .\quad .\quad .\quad \quad .\quad \ . \\ & .\quad \quad .\quad .\quad .\quad \quad .\quad \ . \\ & {{z}_{N1.}}\ \ \ .\quad .\quad {{z}_{Nq}}\quad .\quad {{z}_{Nm}} \\ \end{array}\right. } \right] }_{N\times m}} \end{aligned}$$

The DTBO population is indicated by Z; ${{p}^{th}}$ member of Z is ${{Z}_{p}}$ *i*.*e*. ${{p}^{th}}$ candidate solution of the problem; ${{z}_{pq}}$ is the ${{q}^{th}}$ variable of the ${{p}^{th}}$ solution of the problem;The population size is *N*; No of problem variables is indicated by *m*.

The starting positions of DTBO members (*i*.*e*., potential solutions) are initialized at random at the start of DTBO implementation in the following ways:

32 $$\begin{aligned} {{z}_{pq}}=z_{pq}^{\min }+r*\left( z_{pq}^{\max }-z_{pq}^{\min } \right) \quad for\,p=1\,to\,N\ \varsigma \ q=1\ to\ m \end{aligned}$$

where the upper and lower bounds, respectively, of the ${{q}^{th}}$ variable of the problem under consideration are denoted by $z_{pq}^{\max }$ and $z_{pq}^{\min }$; An unbiased random number between 0 and 1 is denoted by *r*.

The objective function’s value is calculated for each unique candidate solution and is shown as follows:

33 $$\begin{aligned} F={{\left[ {\left\{ \begin{array}{ll} & {{F}_{1}} \\ & . \\ & . \\ & {{F}_{p}} \\ & . \\ & . \\ & {{F}_{N}} \\ \end{array}\right. } \right] }_{N\times 1}}={{\left[ {\left\{ \begin{array}{ll} & F\left( {{Z}_{1}} \right) \\ & \\ & \\ & F\left( {{Z}_{p}} \right) \\ & \\ & \\ & F\left( {{Z}_{N}} \right) \\ \end{array}\right. } \right] }_{N\times 1}} \end{aligned}$$

**Fig. 2:**
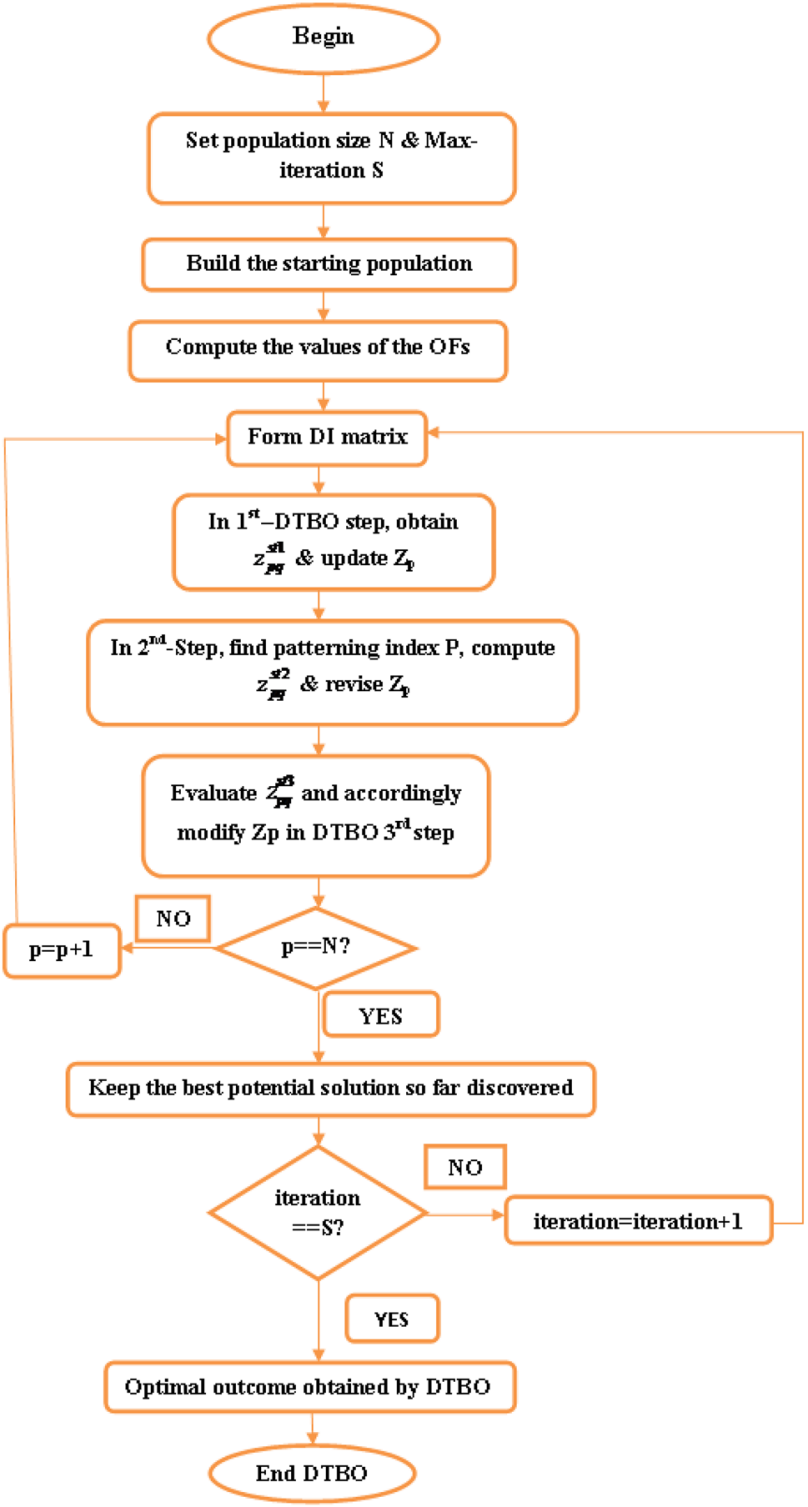
Flowchart of DTBO.

The decisive criterion for evaluating the merits of the solutions under consideration is the computed values of the objective function. The best member is determined by selecting the candidate solution that yields the best value of the objective function. As the iteration moves forward, the top member gets updated. The following three processes make up the process of revising candidate solution in DTBO:

step 1.: **Training by the driving instructor (Exploration):** Few graceful members from DTBO population are chosen to be driving instructors, while the remaining members are categorized as trainee drivers. The capacity to perform a global search to find the optimal solution area for the given problem is accomplished by the skillful selection of instructors and the attaining the instructor’s skill. *L* DTBO members are selected as instructors in each iteration based on a comparison of the objective function values. These members are represented as the driving matrix *DI* in the following manner:
  34 $$\begin{aligned} DI={{\left[ {\left\{ \begin{array}{ll} & D{{I}_{1}} \\ & . \\ & . \\ & D{{I}_{p}} \\ & \\ & D{{I}_{L}} \\ \end{array}\right. } \right] }_{L\times m}}={{\left[ {\left\{ \begin{array}{ll} & D{{I}_{11}}\quad .\quad .\quad D{{I}_{1q}}\quad .\quad D{{I}_{1m}} \\ & .\quad \quad \,\,.\quad \quad \quad .\quad \ \ \ \ .\quad \ \,. \\ & \quad \quad \,\,.\quad \quad \quad .\quad \ \ \ \ .\quad \ \,. \\ & D{{I}_{p1}}\quad .\quad .\quad D{{I}_{pq}}\quad .\quad D{{I}_{pm}}\ \\ & .\quad \quad \,\,.\quad \quad \quad .\quad \ \ \ \ .\quad \ \,. \\ & .\quad \quad \,\,.\quad \quad \quad .\quad \ \ \ \ .\quad \ \,. \\ & D{{I}_{L1.}}\ \ \ .\quad .\quad D{{I}_{Lq}}\quad .\quad D{{I}_{Lm}} \\ \end{array}\right. } \right] }_{L\times m}} \end{aligned}$$
  $D{{I}_{p}}$ is ${p}^{th}$ driving instructor. $D{{I}_{pq}}$ is ${{q}^{th}}$ variable of ${p}^{th}$ instructor.
  35 $$\begin{aligned} L=\left\lfloor 0.1\times N\times \left( \frac{1-s}{S} \right) \right\rfloor \end{aligned}$$
  S is the maximum iteration, while s represents the current iteration. The adjusted position of the DTBO population member is obtained as follows in this step:
  36 $$\begin{aligned} z_{pq}^{st1}={\left\{ \begin{array}{ll} & {{z}_{pq}}+r.\left( D{{I}_{kpq}}-I.{{z}_{pq}} \right) ,\ \ {{F}_{DI{{k}_{p}}}}<{{F}_{p}} \\ & {{z}_{pq}}+r.\left( {{z}_{pq}}-D{{I}_{kpq}} \right) ,\ otherwise \\ \end{array}\right. } \end{aligned}$$
  In the set $\{1,2\}$, *I* represents a random number, and *r* represents a random value between 0 and 1. A random selection of *k* is made from the collection 1,2,...,L in $D{{I}_{kpq}}$ *i*.*e*. ${{k}^{th}}$ driving instructor whose objective function value is ${{F}_{DI{{k}_{p}}}}$, *p* denotes ${{p}^{th}}$ trainee member of the population which is under the training of ${{k}^{th}}$ instructor. When new position provides fitter solution than earlier position then the position is updated by (37).
  37 $$\begin{aligned} {{Z}_{p}}= {\left\{ \begin{array}{ll} & Z_{p}^{st1},\quad F_{p}^{st1}<{{F}_{p}} \\ & {{Z}_{p}},\quad otherwise \\ \end{array}\right. } \end{aligned}$$
  The revised ${{p}^{th}}$ candidate solution at the $1^{st}$ DTBO step is $Z_{p}^{st1}$; $z_{pq}^{st1}$ is its ${{q}^{th}}$ problem variable, The value of its objective function is $F_{p}^{st1}$.
step 2.: **Patterning of the instructor skills of the student driver (Exploration):** In the $2^{nd}$ step, the trainee driver mimics the instructor’s techniques and actions to enhance the DTBO solutions. Members of the DTBO reach a new area of the search space through this procedure. It strengthens DTBO’s exploration power. The DTBO members and instructors combine linearly to form a modified position, which is mathematically represented by (38). If the value of the objective function is better at the new position than it was at the previous one, then (39) is used to replace the previous position.
  38 $$\begin{aligned} & z_{pq}^{st2}=\xi .{{z}_{pq}}+\left( 1-\xi \right) .D{{I}_{kpq}} \end{aligned}$$
  39 $$\begin{aligned} & {{Z}_{p}}={\left\{ \begin{array}{ll} & Z_{p}^{st2}\,\ F_{p}^{st2}<{{F}_{p}} \\ & {{Z}_{p}},\ \ otherwise \\ \end{array}\right. } \end{aligned}$$
  The $Z_{p}^{st2}$ is the updated ${{p}^{th}}$ candidate solution on the DTBO second stage, $z_{pq}^{st2}$ is its ${{q}^{th}}$ variable, The related objective function value is $F_{p}^{st2}$. The patterning index $\xi$ is given by:
  40 $$\begin{aligned} \xi =0.01+0.9\left( 1-\frac{s}{S} \right) \end{aligned}$$
step 3.: **Personal practice (Exploitation):** Based on individual practice, the novice drivers’ driving abilities are improved in this phase. It is akin to exploiting DTBO’s local search capability. Every learner looks for a better position around their existing position. By (41), new positions are generated in close proximity to the existing position. The previous position is replaced by the new one using (42) while it upgrades the objective function value as follows:
  41 $$\begin{aligned} & z_{p,q}^{st3}={{z}_{pq}}+\left( 1-2r \right) .R.\left( 1-\frac{s}{S} \right) .{{z}_{pq}} \end{aligned}$$
  42 $$\begin{aligned} & {{Z}_{p}}={\left\{ \begin{array}{ll} & Z_{p}^{st3},\ F_{p}^{st3}<{{F}_{p}} \\ & {{Z}_{p}},\ otherwise \\ \end{array}\right. } \end{aligned}$$
  $Z_{p}^{st3}$ is modified ${{p}^{th}}$ possible solution at the $3^{rd}$ DTBO phase; $z_{p,q}^{st3}$ is its ${{q}^{th}}$ variable; the value of the related objective function is $F_{p}^{st3}$; *r* is arbitrary quantity, ranging from 0 to 1.; *R* is 0.05, *s* is present iteration & *S* is the maximum iteration. Steps one through three update the DTBO population, completing one DTBO iteration. Then, with a freshly updated population, the subsequent iteration begins and this procedure is ongoing [through (34) to (42)] till the end of the last iteration. The best potential solution is noted as the problem’s solution at the conclusion of the last iteration. Flowchart of DTBO is shown in Fig. 2

## Simulation outcomes & key observations

This section presents the simulation findings for various ORPD case studies using the DTBO algorithm and compares them with the results given in^6^. The entire simulation is run within the MATLAB framework. The selection of test systems includes the conventional IEEE 30, 57,118, 300 bus networks and their modified architectures in modules: one, two, three, four & five. Table 2 provides a brief summary of the test systems under module one and two. Two test networks that are listed in Table 2 are base configuration and adapted configuration. There are five main test modules that comprise the current study. The module three comprises of IEEE 57 bus network. Module four includes IEEE −118 bus system and Module five considers IEEE-300 bus network. In order to provide an impartial comparison, the test systems are selected based on the system utilized in^6^.

**Table 2:** IEEE 30-bus description.

		Base Configuration		Adapted Configuration
Items	Quantity	Details	Quantity	Details
Buses	Thirty	^25^	Thirty	^25^
Branches	Forty-one	^25^	Forty-one	^25^
Thermal generators	Six	$B_1~\text{( }Swing),~B_2,~B_5,~B_8,~B_{11},~ \text {and}~B_{13}$	Three	$B_1~\text{( }Swing),~B_2,~B_8$
WP unit	Nil	-	Two	Bus: Five, Thirteen
Solar PV unit	Nil	-	One	Bus: Eleven
HP unit	Nil	-	one	Bus: Thirteen
Transformer	Four	$L_{6-9},~~L_{6-10},~L_{4-12\;\text {and}~L_{28-27}}$:	Four	$L_{6-9},~~L_{6-10},~L_{4-12\; \text {and}~L_{28-27}}$
Control variables	Nineteen	$V_{TG}$:Six; $TR_{tran}$:Four; $Q_c$:Nine	Nineteen	$V_{TG}$:Three; $V_{WT}$:Two; $V_{PV}$:One;; $TR_{tran}$:Four; $Q_c$:Nine
Load demand	-	283.4MW, 126.2MVAr	-	Same as previous
Range of load bus voltage	Twenty-four	0.95–1.05*p*.*u*.	Twenty Four	0.95–1.05*p*.*u*.
STATCOM	Nil		One	Branch location and rating optimized
		$QC_{10}$, $QC_{12}$, $QC_{15}$, $QC_{17}$, $QC_{20}$,		
Compensation devices	Nine	$QC_{21}$, $QC_{23}$, $QC_{24}$ and $QC_{29}$	Nine	Same as previous

**Fig. 3:**
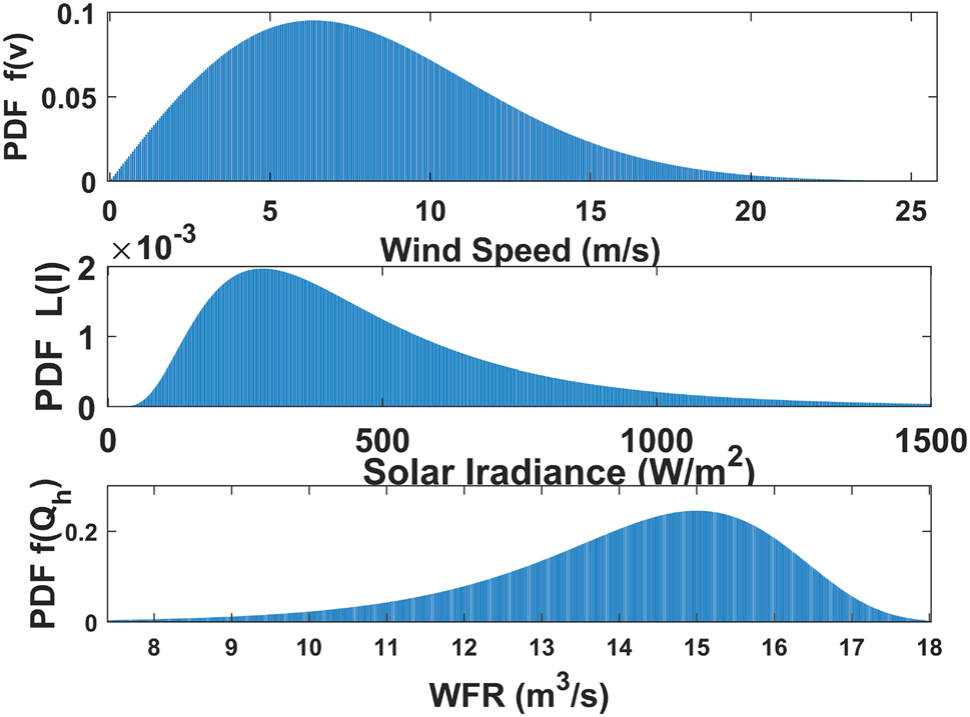
Weibull based WS PDF with $\iota =10$ and $\varkappa =2$, Lognormal based SI $(W/m^2)$ PDF with $\varepsilon =6$ and $\lambda =0.6$ & Gumbel distribution based WFR PDF with location factor 15 & scale factor 1.2.

Only thermal generation is taken into account in test module one, however RESs are added together with an earlier test system in test module two. A total of thirty cases are examined over these five test networks; these are compiled at Table 3. Fig. 3 displays the WS PDF (weibull based), SI PDF (lognormal based), and WFR PDF (Gumbel distribution based) according to the previously indicated parameter values. These are employed to estimate the uncertainty of the RESs.

**Table 3:** Proposed case studies.

Case	Single objective	Multi-objective	Considered objectives	Constraints	Test system
1	$\checkmark$		APL minimization	Equality and Inequality	IEEE 30-Bus
1 A	$\checkmark$		Reactive power loss minimization		
2	$\checkmark$		AVD minimization		
3	$\checkmark$		VSI minimization		
4		$\checkmark$	Simultaneous minimization of APL and AVD		
5	$\checkmark$		APL minimization	Equality and Inequality	IEEE 30-Bus incorporating STATCOM
5 A	$\checkmark$		Reactive power loss minimization		
5B	$\checkmark$		Cost minimization for STATCOM		
6	$\checkmark$		AVD minimization		
7	$\checkmark$		VSI minimization		
8		$\checkmark$	Simultaneous minimization of APL and AVD		
8 A		$\checkmark$	Simultaneous minimization of Active power loss, Voltage deviation and Voltage stability		
9	$\checkmark$		APL minimization	Equality and Inequality	IEEE 30-Bus incorporating wind, PV and hydro energy
9 A	$\checkmark$		Reactive power loss minimization		
10	$\checkmark$		AVD minimization		
11	$\checkmark$		VSI minimization		
12		$\checkmark$	Simultaneous minimization of APL and AVD		
13	$\checkmark$		APL minimization	Equality and Inequality	IEEE 30-Bus incorporating wind, PV, hydro energy and STATCOM
13 A	$\checkmark$		Reactive power loss minimization		
13B	$\checkmark$		Cost minimization for STATCOM		
14	$\checkmark$		AVD minimization		
15	$\checkmark$		VSI minimization		
16		$\checkmark$	Simultaneous minimization of APL and AVD		
17	$\checkmark$		APL minimization	Equality and Inequality	IEEE 57-Bus incorporating wind, PV, hydro energy and STATCOM
18	$\checkmark$		Reactive power loss minimization		
19	$\checkmark$		Cost minimization for STATCOM		
20	$\checkmark$		AVD minimization		
21	$\checkmark$		Voltage stability minimization		
22		$\checkmark$	Simultaneous minimization of Active power loss, Voltage deviation and Voltage stability		
23	$\checkmark$		APL minimization	Equality and Inequality	IEEE 118-Bus incorporating wind, PV, hydro energy and STATCOM
24	$\checkmark$		AVD minimization		
25	$\checkmark$		Voltage stability minimization		
26		$\checkmark$	Simultaneous minimization of Active power loss, Voltage deviation and Voltage stability		
27	$\checkmark$		APL minimization	Equality and Inequality	IEEE 300-Bus incorporating wind, PV, hydro energy and STATCOM
28	$\checkmark$		AVD minimization		
29	$\checkmark$		Voltage stability minimization		
30		$\checkmark$	Simultaneous minimization of Active power loss, Voltage deviation and Voltage stability		

Cases 1 through 8 are examined in test module one, and cases 9 through 16 are investigated in module two. It is possible to split test modules one and two into two categories: those that use STATCOM as a FACTs tool integrated into the test network and those that do not. Cases 1–4 and 9–12 explicitly do not take STATCOM into account, while cases 5–8 and 13–16 are investigated taking STATCOM into account along with a test system.Cases 17 to 22 are taken in Module three. Cases 23 through 26 are considered in Module four & Cases 27 through 30 are under Module five. With the exception of the swing generator, the active power settings for generators in an optimization problem must be carefully selected within the generators’ specific operating parameters. Throughout the course of the study, these amounts are shown for cases 1–8, as well as for cases 9–16, in Table 2. There are different objectives with considered test setting. These are the following: lowering APL, reactive power loss minimization, cost minimization for STATCOM, minimizing AVD and reducing VSI as single objective cases, and concurrently lowering APL and AVD as multi-objective cases.

### Module one

In the left column of Table 2, the test network for this module is provided under the “Base configuration” heading. For this test setup, cases 1 through 8 are run, and for cases 5–8, STATCOM is integrated with the test network. The module has been taken into consideration for a constant 100% network loading. Table 4 and Table 5, respectively, include the computed results for cases 1–4 and cases 5–8. The estimated magnitudes of the objective quantities are displayed in these tables together with the optimal and extreme border values of each variable.

**Table 4:** IEEE 30-bus simulation results for fixed loading (100%).

	Control Parameters	Min.	Max.	Case1^6^	Case1 DTBO	Case1 A	Case2^6^	Case2 DTBO	Case3^6^	Case3 DTBO	Case4 DTBO
Generator voltage (p.u.)	1	0.9	1.1	1.1	1.0771	1.0918	1.0077	1.0195	1.0984	1.0922	1.0346
	2	0.9	1.1	1.0944	1.0653	1.0802	0.9929	1.0133	1.0897	1.0866	1.0338
	5	0.9	1.1	1.075	1.0382	1.0508	1.0691	1.0203	1.0915	1.095	1.0117
	8	0.9	1.1	1.077	1.045	1.0416	1.007	0.9928	1.0903	1.0715	0.988
	11	0.9	1.1	1.1	1.0965	1.0912	0.9973	0.98	1.0999	1.0755	1.0364
	13	0.9	1.1	1.1	1.0914	1.0972	1.0074	1.0356	1.0862	0.9951	1.0251
Transformer tap setting	$\text {Line}_{11}(p.u.)$	0.9	1.1	0.9961	1.004	0.9558	1.0084	0.9994	1.0435	1.0905	1.0343
	$\text {Line}_{12}$	0.9	1.1	0.9027	0.9211	0.9121	0.9022	0.9401	0.9413	1.003	0.9152
	$\text {Line}_{15}$	0.9	1.1	0.9496	0.9801	0.9558	0.9687	1.0885	0.9749	1.0907	1.005
	$\text {Line}_{36}$	0.9	1.1	0.9454	0.9516	0.9048	0.9634	1.0058	0.981	0.9118	0.9456
$Q_{VAr} (MVAR)$	10	0	5	4.986	4.38	4.88	4.501	0.44	4.986	4.25	4.53
	12	0	5	4.927	4.7	4.93	1.691	2.68	4.856	4.51	0.05
	15	0	5	5	4.71	4.92	4.268	4.33	4.549	4.29	3.51
	17	0	5	4.881	4.79	4.73	0.38	2.53	4.988	4.87	0.04
	20	0	5	4.648	4.79	4.62	4.918	4.99	4.983	0.55	4.97
	21	0	5	4.986	4.76	4.82	4.33	4.6	4.776	2.97	4.92
	23	0	5	4.114	2.52	4.93	4.869	2.32	4.996	2.43	4.78
	24	0	5	4.95	4.38	4.67	4.89	3.81	4.835	4.48	4.95
	29	0	5	2.146	2.66	4.74	1.509	2.96	4.766	4.99	0.78
APL(MW)				**4.5086**	**4.3101**	4.48	5.7777	5.26	4.7272	5.8011	**5.09**
Qloss(MVAr)				NA	NA	−70.35	NA	NA	NA	NA	NA
AVD(p.u.)				2.3375	1.6068	2.2585	**0.0879**	**0.0794**	2.082	1.4756	**0.1276**
VSI(p.u.)				0.1119	0.13	0.12	0.1371	0.1387	**0.1132**	**0.1104**	0.1477
Reactive power generation (MVAr)	1	−20	150	NA	2.88	8.19	NA	−14.64	NA	−15.67	−16.17
	2	−20	60	NA	4.81	21.73	NA	−8.14	NA	−12.93	41.15
	5	−15	62.5	NA	19.28	24.25	NA	53.7	NA	52.17	34.41
	8	−15	48.7	NA	21.2	10.39	NA	9.07	NA	9.31	0.93
	11	−10	40	NA	12.24	−3.62	NA	−7.21	NA	20.32	17.02
	13	−15	44.7	NA	5.93	−5.09	NA	24.66	NA	−13.28	11.4
CPU Time(s)				128.75	123.83	127.452	130.33	129.87	127.13	122.76	121.98

**Table 5:** IEEE 30-bus simulation results for fixed loading (100%) with STATCOM.

	Control Parameters	Min.	Max.	90 Case 5 DTBO	Case 5 A	Case 5B	Case 6 (DTBO)	Case 7 (DTBO)	Case 8 DTBO	Case 8 A
Generator voltage (p.u.)	1	0.95	1.1	1.0676	1.0649	1.0914	1.017	1.0892	1.0301	1.0366
	2	0.95	1.1	1.0561	1.0555	1.0746	1.014	1.0728	1.0223	1.0324
	5	0.95	1.1	1.0335	1.0291	1.04	1.0162	1.0316	1.0094	1.0098
	8	0.95	1.1	1.0418	1.0219	1.0376	0.9952	1.079	0.9819	0.9962
	11	0.95	1.1	1.0986	1.0966	1.0822	1.0096	1.0867	1.0415	1.0557
	13	0.95	1.1	1.0996	1.0919	1.0954	0.9979	1.0507	1.0228	1.0252
Transformer tap setting		0.9	1.1	0.9812	0.9046	0.9516	0.9987	1.0959	1.0353	1.0517
		0.9	1.1	0.902	0.9088	0.9151	1.0569	1.0055	0.9488	0.9266
		0.9	1.1	0.9229	0.9234	0.9368	0.9808	1.0267	1.0178	1.0035
		0.9	1.1	0.9267	0.9114	0.9002	0.9971	0.9002	0.9549	0.9759
$$Q_{VAr}$$(MVAr)	10	0	5	4.95	4.95	4.93	4.72	4.67	1.94	3.89
	12	0	5	4.95	4.88	4.87	3.36	4.98	0.62	0.44
	15	0	5	2.65	4.99	4.9	2.35	3.16	2.13	2.77
	17	0	5	3.12	5	4.69	3.02	4.61	1.32	0.25
	20	0	5	1.76	4.97	4.86	4.64	4.99	4.11	4.97
	21	0	5	4.71	4.85	4.95	4.65	4.77	3.69	4.7
	23	0	5	0.7	4.77	4.33	2.5	4.67	3.06	4.88
	24	0	5	2.79	4.94	4.91	4.98	4.38	4.23	4.84
	29	0	5	0.95	4.81	4.93	2.31	4.27	1.5	4.75
Optimal location				26	26	23	26	7	23	**23**
		0.95	1.1	1.0979	1.005	1.054	1.0254	1.0483	0.9994	0.9786
		−20	0	−2.54	−1.765	−2.034	−4.65	−3.76	−3.876	−4.453
APL(MW)				**4.27**	4.54	4.55	5.1102	5.63	5.0701	**4.89**
Qloss(MVAr)				NA	**−69.541**	−69.691	NA	NA	NA	−40.61
Cost of STATCOM ($/h)				15.56	16.77	**15.04**	17.56	15.67	17.56	17.78
AVD(p.u.)				2.2019	2.0971	2.2131	**0.0731**	2.1915	**0.1221**	**0.1355**
VSI(p.u.)				0.1144	0.1239	0.1203	0.1384	**0.1045**	0.1401	**0.1488**
Reactive power generation (MVAr)	1	−20	150	2.11	2.72	20.85	−17.13	5.67	−4.2	−11.07
	2	−20	60	1.3	24.02	15.5	5.23	−19.52	14.38	27.2
	5	−15	62.5	22.24	27.28	19.03	48.66	−4.71	39.7	29.78
	8	−15	48.7	27.38	20.59	15.51	13.74	37.56	−7.54	3.57
	11	−10	40	2.11	−7.64	−6.15	3.58	17.17	19.62	25.71
	13	−15	44.7	−12.73	−10.27	−8.23	−6.73	−13.41	11.98	10.4
	CPU Time (s)			122.76	123.67	124.65	128.21	121.87	121.11	122.45

**Table 6:** Twenty five scenarios based on probable wind speed, solar irradiance and water flow rate and % of loading.

Scenario no.	%Loading	Wind Farm$_1$ at bus 5 WS(m/s)	Solar PV at bus 11 SI $(W/{m}^{2})$	Wind Farm$_2$and HP at bus 13		Scenario probability
				WS (m/s)	WFR$(m^3/s)$	
1	85.4998	11.5643	454.2837	6.7654	13.8877	0.005
2	93.0663	1.9876	783.4354	8.6512	12.5432	0.001
3	84.2514	8.9876	897.8735	6.7809	13.6644	0.004
4	76.5664	3.6543	305.5632	11.8765	11.5432	0.003
5	80.3714	4.6543	289.6734	1.9876	16.0987	0.004
6	98.3753	12.8765	1055.3243	23.7654	15.4321	0.005
7	87.0929	19.5412	987.7676	13.8765	11.3421	0.001
8	88.9426	9.2098	89.7655	0.9876	15.5432	0.04
9	92.3917	3.9861	0	5.003	15.3342	0.011
10	110.5316	15.8702	107.6754	4.7865	14.6565	0.005
11	81.5345	14.731	755.8765	7.1122	11.6543	0.001
12	76.5164	7.7654	201.3321	7.9832	10.7687	0.081
13	92.7455	8.8965	95.8908	11.345	14.7765	0.009
14	77.6634	11.8743	1004.5634	4.4321	14.5434	0.002
15	71.8947	5.8667	432.4454	12.865	15.4409	0.001
16	106.8372	2.8751	212.7609	0.9876	15.8976	0.076
17	68.1133	17.8764	454.5645	3.872	13.6543	0.3
18	74.0076	12.5436	390.8981	8.3241	11.3342	0.049
19	97.2919	11.8043	107.9891	6.3421	12.1213	0.001
20	71.5136	6.765	0	4.4531	13.3423	0.211
21	100.2235	4.6754	234.8976	17.8711	13.098	0.077
22	86.3776	7.2342	243.0098	3.9861	16.6548	0.046
23	78.5634	9.8877	465.9878	4.2324	16.3377	0.05
24	78.5748	12.541	777.9872	16.321	10.9899	0.001
25	84.8562	3.4532	867.1102	5.876	12.6544	0.016

**Table 7:** Output power of different generators for different loading.

Scenario no.	%Loading	Wind Farm$_1$ at bus 5	Solar PV at bus 11	Wind Farm$_2$ at bus 13	Hydro power at bus 13	Wind Farm$_2$+Hydro at bus 13	Scenario probability
		WP1(MW)	PV power (MW)	WP2(MW)	HP(MW)	HP+WP2 (MW)	
1	85.4998	49.4094	22.7142	13.0341	2.8951	15.9291	0.005
2	93.0663	0	39.1718	19.5618	2.6148	22.1766	0.001
3	84.2514	34.5438	44.8937	13.0877	2.8485	15.9362	0.004
4	76.5664	3.7748	15.2782	30.7263	2.4063	33.1327	0.003
5	80.3714	9.544	14.4837	0	3.356	3.356	0.004
6	98.3753	56.9798	50	45	3.217	48.217	0.005
7	87.0929	75	49.3884	37.6494	2.3644	40.0138	0.001
8	88.9426	35.8258	3.3574	0	3.2402	3.2402	0.04
9	92.3917	5.689	0	6.9335	3.1966	10.1301	0.011
10	110.5316	74.2512	4.8308	6.184	3.0553	9.2394	0.005
11	81.5345	67.6788	37.7938	14.2345	2.4295	16.664	0.001
12	76.5164	27.4927	10.0666	17.2495	2.2449	19.4944	0.081
13	92.7455	34.0183	3.8313	28.8865	3.0803	31.9669	0.009
14	77.6634	51.1979	50	4.9573	3.0318	7.989	0.002
15	71.8947	16.5387	21.6223	34.1481	3.2188	37.3669	0.001
16	106.8372	0	10.638	0	3.3141	3.3141	0.076
17	68.1133	75	22.7282	3.0185	2.8464	5.8649	0.3
18	74.0076	55.0592	19.5449	18.4296	2.3628	20.7923	0.049
19	97.2919	50.794	4.859	11.5688	2.5268	14.0956	0.001
20	71.5136	21.7212	0	5.03	2.7814	7.8113	0.211
21	100.2235	9.6658	11.7449	45	2.7304	47.7304	0.077
22	86.3776	24.4281	12.1505	3.4134	3.4719	6.8853	0.046
23	78.5634	39.7367	23.2994	4.266	3.4058	7.6718	0.05
24	78.5748	55.0442	38.8994	45	2.291	47.291	0.001
25	84.8562	2.6146	43.3555	9.9554	2.638	12.5934	0.016

The modified artificial hummingbird algorithm (MAHA) used in^6^ is used as a comparable test setup. The DTBO algorithm is being used in this work to reduce APL, AVD, VSI as single OFs and to reduce APL and AVD at the same time.

From Table 4, crucial observations are:

- APL is determined as 4.3101 (MW) in case 1 using DTBO, whereas it was 4.5086 (MW) in^6^. Therefore, DTBO lowers APL in relation to^6^ by 0.1985(MW).
- Using DTBO, the AVD in case-2 is 0.0794 *p*.*u*., which is less than the AVD found in^6^ by 0.0085 *p*.*u*.
- With DTBO, the VSI in case-3 is 0.1104, whereas in^6^, it was 0.1132. DTBO hence lowers VSI by 0.0028 as opposed to^6^.
- The outcomes in case 4 are noteworthy when simultaneous aims, namely APL & AVD, are taken into account. Case 4’s APL and AVD are both higher than Case 1 and Case 2, respectively, but taken as a whole, APL & AVD are better than Case 1 or Case 2.

The final row of this result lists the computational duration for each case. It indicates that, when compared to^6^, using DTBO not only improves the optimization results for cases 1–3, but also helps to occupying better results in a shorter amount of time.

The results of the experiments that were carried out while taking into account STATCOM with base configuration are shown in Table 5, which reveals that:

- The computed APL value in case 5 is 4.27(MW), which is less than 0.0401(MW) from the APL of case 1.
- The calculated AVD in case 6 is 0.0731(*p*.*u*.). The AVD, found in case 2 is higher than the AVD of case 6 by 0.0063(*p*.*u*.).
- The calculated VSI for case 7 is 0.1045(*p*.*u*.). As for case 7, the VSI is lower by 0.0059(*p*.*u*.) than the VSI obtained in case 3.
- APL & AVD in the multi-objective situation (case 8) are 5.0701(MW) & 0.1221 (*p*.*u*.), respectively, which are better than those figures in case 4.
- As previously stated, the test configuration that was used for cases 1–4, has been altered by adding STATCOM, and cases 5–8 have been resolved using this updated setup.

**Fig. 4:**
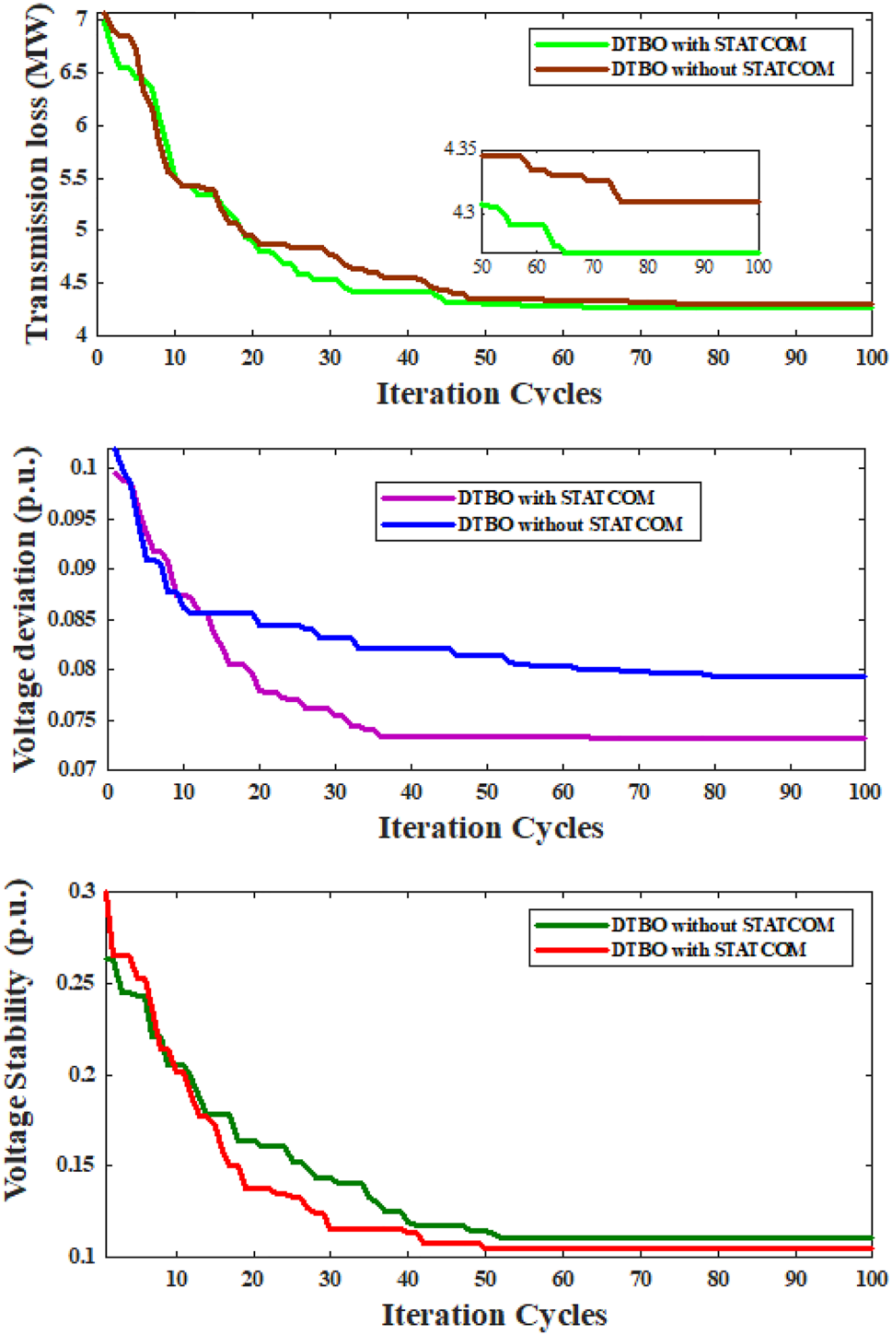
Using DTBO with and without STATCOM, the convergence characteristics of APL, AVD & VSI.

The aforementioned findings (cases 1 through case 8)make it abundantly evident that the success of the ORPD issue is greatly aided by the use of STATCOM in the power network. Fig. 4 provides the convergence characteristics of APL minimization, AVD minimization & VSI minimization with and without consideration of STATCOM. From the curves in Fig. 4, it is clear that adding STATCOM to the power network improved system performances.

### Module two

As indicated in Table 3, eight examples (cases 9–16) are taken into consideration in this phase of the experiment, the first four cases (cases 9–12) are carried out using a test setup without the introduction of STATCOM, and the remaining cases (cases 13–16) are carried out over a test network that has STATCOM. Table 2’s right portion displays the test configuration that was used for this part of study. This type of adjustment, referred as “adapted configuration,” involves combining a conventional model with RESs (WP, PV & HP). Furthermore, in this experimental mode, the process of scenario generation and scenario downsizing has been utilized to address the volatility of RESs and the unpredictability of load^25^. For estimating variable load demands, an average PDF with mean=70 and standard deviation=10 has been considered^25^. Weibull, lognormal, and gumbel PDFs are used to model uncertain WS, SI, and WFR, respectively, during the scenario design process. During the construction of the scenarios, nil irradiance is assigned with 50% chance because the sun is present for just about half of a 24-hour day. The remaining 50% of the possibilities are allocated with non-zero PV power contribution to the scenarios. The load demand, WS, SI, and WFR are the elements of a single scenario.

To begin with, the 1000 Monte-Carlo options for load demand, WS, SI, and WFR are combined to create a set of 1000 scenarios. The 1000 situations have been reduced to 25 scenarios by BRA^65^ since handling 1000 possibilities is not manageable. Initially, $N_{0}$ scenarios are considered where each of them having probabilities of (${{\rho }_{0}}=\frac{1}{N_0}$). After every BRA iteration, one scenario is removed in an effort to reduce the total number of possibilities. The following are the steps that the BRA takes to reduce scenarios: 

1. **Initialization**

- Create $N_{0}$ scenarios ($S_{i}$ for $i= 1, 2, .~.~., N_{0}$). Currently: $N_{0} = 1000$.
- At the beginning, the chance of every scenario is identical (${{\rho }_{0}}=\frac{1}{N_0}$). Determine the distance $d_{ij}$ among each pair of scenarios. where ${{d}_{ij}}=\left\| {{S}_{i}}-{{S}_{j}} \right\|$.
- With $d_{ij}$, set up distance matrix *D* with starting dimension ${{N}_{0}}\times {{N}_{0}}$ and diagonal elements $d_{ii}=0$.
- Allot a running variable $N_r = N_{0}$ & stopping criterion $N_{ec}$, indicates the count of final preferred scenarios.

2. **Looping events**

step 1.: Find least distance value (apart from self-distance $d_{ii}=0$) from *D*. Suppose $d_{mn}$ is least in *D* (*i*.*e*. separation between $m^{th}$ and $n^{th}$ scenarios), and suppose scenarios $S_{m} and S_{n}$ having likelihoods of $\rho _{m}$ and $\rho _{n}$ respectively.
step 2.: If ${{\rho }_{m}}\ge {{\rho }_{n}}$, remove scenario *n*. Modify likelihood ${{\rho }_{m}}={{\rho }_{m}}+{{\rho }_{n}}$. Else, take away scenario *m*. Alter probability ${{\rho }_{n}}={{\rho }_{m}}+{{\rho }_{n}}$.
step 3.: Allocate ${{N}_{r}}={{N}_{r}}-1$. reassess the matrix *D*, composed of distance between each pair of existing scenarios.
step 4.: If ${{N}_{r}}>{{N}_{ec}}$, jump to STEP 1 of reiterating. Else, END.

These 25 scenarios, together with their associated possibilities, are displayed in Table 6 and are generated by applying BRA to 1000 initial scenarios. The load demand is presented in Table 6 as % loading. From the scenarios given in Table 6 and through equations (5), (10) and (12), respectively, the corresponding WP, PV & HP are evaluated and shown in Table 7.

The optimization algorithm is then executed over every scenario independently. The results of running those algorithms are the OFs, which are the multi-objective minimization of combined APL and AVD and the single-objective minimization of APL, AVD, and VSI. As the current study consists of 25 situations, the optimization technique is run 25 times to cover all the developed scenarios in order to thoroughly investigate any case.

For each constructed scenario in Table 6, Table 8 displays the minimal APL (corresponds to case 9), minimum AVD (corresponds to case 10), and minimum VSI (corresponds to case 11). An expected APL (EAPL) (for case 9), an expected reactive power loss (ERPL) (in case 9 A), an expected AVD (EAVD) (for case 10), and an expected VSI (EVSI) (for case 11) are computed and reported in Table 8 from these probable computed APL, AVD, and VSI for each scenario. These calculations are done as follows:

43 $$\begin{aligned} & EAPL=\sum \limits _{i=1}^{{{N}_{ec}}}{{{\rho }_{i}}\times APL_{i}} \end{aligned}$$

44 $$\begin{aligned} & EAVD=\sum \limits _{i=1}^{{{N}_{ec}}}{{{\rho }_{i}}\times AVD_{i}} \end{aligned}$$

45 $$\begin{aligned} & EVSI=\sum \limits _{i=1}^{{{N}_{ec}}}{{{\rho }_{i}}\times VSI_{i}} \end{aligned}$$

46 $$\begin{aligned} & ERPL=\sum \limits _{i=1}^{{{N}_{ec}}}{{{\rho }_{i}}\times RPL_{i}} \end{aligned}$$

where: *i* indicates scenario index; $N_{ec}$ is the number of scenario; $\rho _{i}$ indicates probability of $i^{th}$ scenario.

**Table 8:** Single-objective ORPD solution of IEEE 30 bus with RESs.

Scenario no.	%Loading	Wind Farm$_1$ at bus 5	Solar PV at bus 11	Wind Farm$_2$ at bus 13	Hydro power at bus 13	Wind Farm$_2$+Hydro at bus 13	Scenario probability	Scenario- based APL	Scenario- based Qloss	Scenario- based AVD	Scenario- based VSI
		WP$_1$(MW)	PV (MW)	WP$_2$(MW)	HP(MW)	HP+WP$_2$(MW)	$\Delta$sc	(MW)	(MVAR)	(*p*.*u*.)	(*p*.*u*.)
1	85.4998	49.4094	22.7142	13.0341	2.8951	15.9291	0.005	2.3411	−85.450	0.0711	0.0987
2	93.0663	0	39.1718	19.5618	2.6148	22.1766	0.001	2.1121	−63.560	0.0638	0.0921
3	84.2514	34.5438	44.8937	13.0877	2.8485	15.9362	0.004	2.6402	−73.674	0.0792	0.0765
4	76.5664	3.7748	15.2782	30.7263	2.4063	33.1327	0.003	3.1198	−45.564	0.068	0.0857
5	80.3714	9.544	14.4837	0	3.356	3.356	0.004	1.9743	−35.674	0.0756	0.0884
6	98.3753	56.9798	50	45	3.217	48.217	0.005	1.7578	−81.674	0.0436	0.0812
7	87.0929	75	49.3884	37.6494	2.3644	40.0138	0.001	1.9645	−64.657	0.0711	0.0798
8	88.9426	35.8258	3.3574	0	3.2402	3.2402	0.04	1.4372	−77.894	0.0528	0.0901
9	92.3917	5.689	0	6.9335	3.1966	10.1301	0.011	2.0976	−67.910	0.0707	0.0786
10	110.5316	74.2512	4.8308	6.184	3.0553	9.2394	0.005	3.9543	−45.674	0.0727	0.0854
11	81.5345	67.6788	37.7938	14.2345	2.4295	16.664	0.001	1.3202	−82.674	0.0648	0.0965
12	76.5164	27.4927	10.0666	17.2495	2.2449	19.4944	0.081	1.2263	−38.785	0.0562	0.0876
13	92.7455	34.0183	3.8313	28.8865	3.0803	31.9669	0.009	3.4409	−64.229	0.0726	0.0811
14	77.6634	51.1979	50	4.9573	3.0318	7.989	0.002	3.5234	−47.785	0.0781	0.0912
15	71.8947	16.5387	21.6223	34.1481	3.2188	37.3669	0.001	4.2998	−39.785	0.0744	0.0765
16	106.8372	0	10.638	0	3.3141	3.3141	0.076	3.4532	−42.783	0.0704	0.0729
17	68.1133	75	22.7282	3.0185	2.8464	5.8649	0.3	1.9225	−38.748	0.0543	0.0897
18	74.0076	55.0592	19.5449	18.4296	2.3628	20.7923	0.049	1.5243	−73.748	0.0676	0.0786
19	97.2919	50.794	4.859	11.5688	2.5268	14.0956	0.001	5.5521	−80.843	0.0675	0.0902
20	71.5136	21.7212	0	5.03	2.7814	7.8113	0.211	4.6334	−65.839	0.0697	0.0876
21	100.2235	9.6658	11.7449	45	2.7304	47.7304	0.077	3.4477	−46.784	0.0643	0.0765
22	86.3776	24.4281	12.1505	3.4134	3.4719	6.8853	0.046	2.5501	−55.221	0.0763	0.0861
23	78.5634	39.7367	23.2994	4.266	3.4058	7.6718	0.05	1.3392	−59.839	0.0603	0.0911
24	78.5748	55.0442	38.8994	45	2.291	47.291	0.001	1.2906	−44.578	0.0486	0.0817
25	84.8562	2.6146	43.3555	9.9554	2.638	12.5934	0.016	1.7545	−79.895	0.0751	0.0856
**Case 9**		**EAPL**=$\sum \Delta$**sc**$\cdot$**APL**						**2.6719**			
**Case 9 A**		**EQL=**$${\sum \Delta }$$sc$$\cdot$$$$Q_{loss}$$						**−52.4866**			
**Case10**		** EAVD**=$\sum \Delta$**sc**$\cdot$**AVD**						**0.0627**			
**Case11**		** EVSI**=$\sum \Delta$**sc**$\cdot$**VSI**						**0.0858**

**Table 9:** Multi-objective ORPD solution of IEEE 30 bus with RESs.

Scenario no.	%Loading	Wind Farm$$_1$$ at bus 5	Solar PV at bus 11	Wind Farm$$_2$$ at bus 13	Hydro power at bus 13	Wind Farm$$_2+$$ Hydro at bus 13 HP+WP$$_2$$	Scenario probability	Scenario- based APL	Scenario- based AVD	Objective value LVD (Case 12) $$\lambda _l$$$$~~~~~~\cdot$$APL+$$\lambda _{vd}$$$$\cdot$$AVD
		WP$_1$(MW)	PV (MW)	WP$_2$(MW)	HP(MW)	(MW)	$\Delta$sc	(MW)	(*p*.*u*.)	
1	85.4998	49.4094	22.7142	13.0341	2.8951	15.9291	0.005	2.9872	0.0787	3.7742
2	93.0663	0	39.1718	19.5618	2.6148	22.1766	0.001	3.0987	0.0765	3.8637
3	84.2514	34.5438	44.8937	13.0877	2.8485	15.9362	0.004	3.3424	0.0834	4.1764
4	76.5664	3.7748	15.2782	30.7263	2.4063	33.1327	0.003	4.2311	0.0721	4.9521
5	80.3714	9.544	14.4837	0	3.356	3.356	0.004	2.6756	0.0756	3.4316
6	98.3753	56.9798	50	45	3.217	48.217	0.005	2.6546	0.0543	3.1976
7	87.0929	75	49.3884	37.6494	2.3644	40.0138	0.001	2.5455	0.0787	3.3325
8	88.9426	35.8258	3.3574	0	3.2402	3.2402	0.04	2.4372	0.0676	3.1132
9	92.3917	5.689	0	6.9335	3.1966	10.1301	0.011	3.0566	0.0799	3.8556
10	110.5316	74.2512	4.8308	6.184	3.0553	9.2394	0.005	4.7543	0.0801	5.5553
11	81.5345	67.6788	37.7938	14.2345	2.4295	16.664	0.001	2.0202	0.0765	2.7852
12	76.5164	27.4927	10.0666	17.2495	2.2449	19.4944	0.081	2.2263	0.0643	2.8693
13	92.7455	34.0183	3.8313	28.8865	3.0803	31.9669	0.009	3.9909	0.0788	4.7789
14	77.6634	51.1979	50	4.9573	3.0318	7.989	0.002	4.5234	0.0843	5.3664
15	71.8947	16.5387	21.6223	34.1481	3.2188	37.3669	0.001	5.5541	0.0832	6.3861
16	106.8372	0	10.638	0	3.3141	3.3141	0.076	3.9992	0.0787	4.7862
17	68.1133	75	22.7282	3.0185	2.8464	5.8649	0.3	2.9225	0.0675	3.5975
18	74.0076	55.0592	19.5449	18.4296	2.3628	20.7923	0.049	2.4243	0.0776	3.2003
19	97.2919	50.794	4.859	11.5688	2.5268	14.0956	0.001	6.7721	0.0765	7.5371
20	71.5136	21.7212	0	5.03	2.7814	7.8113	0.211	5.7714	0.0803	6.5744
21	100.2235	9.6658	11.7449	45	2.7304	47.7304	0.077	3.9987	0.0765	4.7637
22	86.3776	24.4281	12.1505	3.4134	3.4719	6.8853	0.046	2.9871	0.0799	3.7861
23	78.5634	39.7367	23.2994	4.266	3.4058	7.6718	0.05	2.7787	0.0707	3.4857
24	78.5748	55.0442	38.8994	45	2.291	47.291	0.001	2.4531	0.0611	3.0641
25	84.8562	2.6146	43.3555	9.9554	2.638	12.5934	0.016	2.7865	0.0823	3.6095
		** EAPL**=$\sum \Delta$**sc**$\cdot$**APL**= **3.6137**					$\lambda _l$=1			
**Case-12**		**EAVD**=$\sum \Delta$**sc**$\cdot$**AVD=** **0.0734**					$\lambda _{vd}$=10			

To display the outcomes of case 12, where the target is simultaneously minimize the APL and the AVD for 25 scenarios of Table 6, Table 9 is created.

Experiments for cases 13–16 are carried out on the test setup with STATCOM under the identical scenarios as listed in Table 10. Table 10 contains scenario-based experimental results for instances 13–15, whereas Table 11 has results for case 16. Applying DTBO to test systems with and without STATCOM, the obtained results indicate that:

- Without STATCOM, EAPL was 2.6719 MW (case 9), but with STATCOM, it decreases to 2.5426 MW (case 13).
- The EAVD in case 10 was 0.0627 *p*.*u*. without STATCOM, while in case 14 (which includes STATCOM), it decreases to 0.0596 *p*.*u*..
- Without STATCOM, the EVSI in case 11 was 0.0858 *p*.*u*.; with STATCOM included, the EVSI drops to 0.0818 *p*.*u*. in case 15.
- When case-12 and case-16 are observed simultaneously, it is discovered that, in contrast to case-12 (*i*.*e*. without STATCOM), connecting STATCOM (in case-16) lowers EAPL and EAVD by 0.1974 MW and 0.0035 *p*.*u*., respectively.

These findings imply that to minimize individually APL, AVD, VSI as well as to reduce jointly APL and AVD utilization of STATCOM devices offers positive impacts in the operations of power networks.

**Table 10:** Single-objective ORPD solution of IEEE 30 bus with RESs-STATCOM.

Scenario no.	%Loading	Wind Farm$$_1$$ at bus 5	Solar PV at bus 11	Wind Farm$$_2$$ at bus 13	Hydro power at bus 13	Wind Farm$$_2+$$ Hydro at bus 13 HP+WP$$_2$$	Scenario probability	Scenario- based APL	Scenario- based$$Q_{loss}$$	Scenario- based AVD	Scenario -based VSI
		WP$$_1$$(MW)	PV (MW)	WP$$_2$$(MW)	HP(MW)	$$Q_{loss}$$	(MW)	$$\Delta$$sc	(MW)	(*p*.*u*.)	(*p*.*u*.)
1	85.4998	49.4094	22.7142	13.0341	2.8951	15.9291	0.005	2.1091	−83.991	0.0702	0.0887
2	93.0663	0	39.1718	19.5618	2.6148	22.1766	0.001	1.9821	−57.675	0.0564	0.0876
3	84.2514	34.5438	44.8937	13.0877	2.8485	15.9362	0.004	2.1107	−72.783	0.0756	0.0701
4	76.5664	3.7748	15.2782	30.7263	2.4063	33.1327	0.003	2.1558	−44.673	0.0544	0.0812
5	80.3714	9.544	14.4837	0	3.356	3.356	0.004	1.7653	−35.001	0.0654	0.0809
6	98.3753	56.9798	50	45	3.217	48.217	0.005	1.4532	−79.893	0.0332	0.0787
7	87.0929	75	49.3884	37.6494	2.3644	40.0138	0.001	1.7864	−61.893	0.0687	0.0722
8	88.9426	35.8258	3.3574	0	3.2402	3.2402	0.04	1.3332	−69.9921	0.0476	0.0876
9	92.3917	5.689	0	6.9335	3.1966	10.1301	0.011	2.0043	−66.893	0.0689	0.0745
10	110.5316	74.2512	4.8308	6.184	3.0553	9.2394	0.005	3.7659	−43.849	0.0711	0.0824
11	81.5345	67.6788	37.7938	14.2345	2.4295	16.664	0.001	1.1122	−80.387	0.0623	0.0911
12	76.5164	27.4927	10.0666	17.2495	2.2449	19.4944	0.081	1.1067	−32.998	0.0534	0.0833
13	92.7455	34.0183	3.8313	28.8865	3.0803	31.9669	0.009	3.3429	−63.894	0.0712	0.0766
14	77.6634	51.1979	50	4.9573	3.0318	7.989	0.002	3.3421	−44.785	0.0776	0.0854
15	71.8947	16.5387	21.6223	34.1481	3.2188	37.3669	0.001	4.1987	−39.002	0.0706	0.0709
16	106.8372	0	10.638	0	3.3141	3.3141	0.076	3.3421	−41.092	0.0678	0.0701
17	68.1133	75	22.7282	3.0185	2.8464	5.8649	0.3	1.7864	−37.984	0.0505	0.0854
18	74.0076	55.0592	19.5449	18.4296	2.3628	20.7923	0.049	1.3421	−70.189	0.0624	0.0734
19	97.2919	50.794	4.859	11.5688	2.5268	14.0956	0.001	5.3344	−75.948	0.0655	0.0876
20	71.5136	21.7212	0	5.03	2.7814	7.8113	0.211	4.4987	−64.894	0.0677	0.0833
21	100.2235	9.6658	11.7449	45	2.7304	47.7304	0.077	3.3987	−45.784	0.0621	0.0721
22	86.3776	24.4281	12.1505	3.4134	3.4719	6.8853	0.046	2.4538	−54.784	0.0745	0.0855
23	78.5634	39.7367	23.2994	4.266	3.4058	7.6718	0.05	1.2007	−58.843	0.0573	0.0885
24	78.5748	55.0442	38.8994	45	2.291	47.291	0.001	1.2675	−42.894	0.0477	0.0798
25	84.8562	2.6146	43.3555	9.9554	2.638	12.5934	0.016	1.6875	−75.784	0.0701	0.0814
**Case 13: EAPL**=$$\sum \Delta$$**sc**$$\cdot$$**APL**						**2.5426**					
**Case 13 A: EQL=**$$\sum \Delta$$**sc**$$\cdot Q_{loss}$$						**−50.6848**					
**Case 13B: Cost of STATCOM**						**20.56($/h)**					
**Case14: EAVD**=$$\sum \Delta$$**sc**$$\cdot$$**AVD**						**0.0596**					
**Case15: EVSI**=$$\sum \Delta$$**sc**$$\cdot$$**VSI**						**0.0818**					

**Fig. 5:**
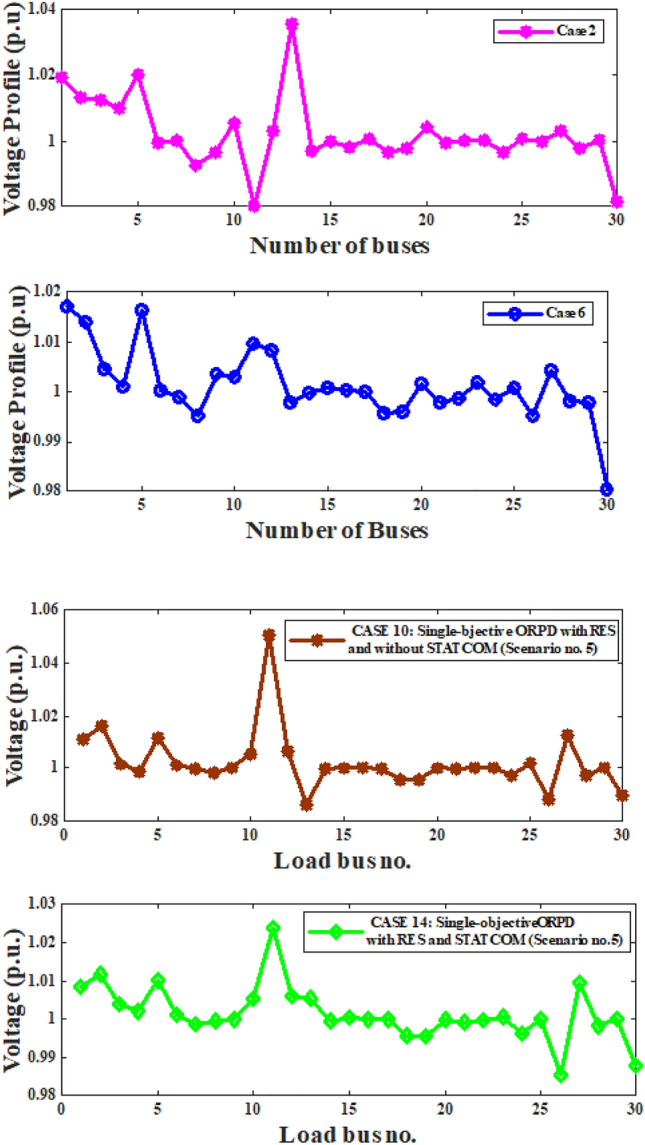
Load bus voltage deviations for case-2, 6, 10 & 14.

Fig. 5 depicts the deviation of voltage on different load buses for case 2 (on only traditional IEEE 30 bus set up), case 6(traditional network with STATCOM), case 10 (RESs included in conventional IEEE 30 bus network) and case 14 (Both RESs and STATCOM included). In these four cases objective was reduction on AVD. It can be observed from the Fig. 5 that the spread of voltage deviation is reducing when RESs & STATCOM devices are being introduced with conventional standard 30 bus network.

**Table 11:** Multi-objective ORPD solution of IEEE 30 bus with RESs-STATCOM.

Scenario no.	%Loading	Wind Farm$$_1$$ at bus 5	Solar PV at bus 11	Wind Farm$$_2$$ at bus 13	Hydro power at bus 13	Wind Farm$$_2+$$ Hydro at bus 13 HP+WP$$_2$$	Scenario probability	Scenario- based APL	Scenario- based AVD	Objective value LVD (Case 16) $$\lambda _l$$$$\cdot$$APL+$$\lambda _{vd}$$$$\cdot$$AVD
		WP$_1$(MW)	PV (MW)	WP$_2$(MW)	HP(MW)	(MW)	$\Delta$sc	(MW)	(*p*.*u*.)	
1	85.4998	49.4094	22.7142	13.0341	2.8951	15.9291	0.005	2.5643	0.0747	3.3113
2	93.0663	0	39.1718	19.5618	2.6148	22.1766	0.001	2.9967	0.0733	3.7297
3	84.2514	34.5438	44.8937	13.0877	2.8485	15.9362	0.004	3.1211	0.0812	3.9331
4	76.5664	3.7748	15.2782	30.7263	2.4063	33.1327	0.003	4.1109	0.0701	4.8119
5	80.3714	9.544	14.4837	0	3.356	3.356	0.004	2.4321	0.0699	3.1311
6	98.3753	56.9798	50	45	3.217	48.217	0.005	2.4434	0.0522	2.9654
7	87.0929	75	49.3884	37.6494	2.3644	40.0138	0.001	2.2212	0.0744	2.9652
8	88.9426	35.8258	3.3574	0	3.2402	3.2402	0.04	2.3342	0.0622	2.9562
9	92.3917	5.689	0	6.9335	3.1966	10.1301	0.011	2.9984	0.0755	3.7534
10	110.5316	74.2512	4.8308	6.184	3.0553	9.2394	0.005	4.5653	0.0765	5.3303
11	81.5345	67.6788	37.7938	14.2345	2.4295	16.664	0.001	1.9875	0.0732	2.7195
12	76.5164	27.4927	10.0666	17.2495	2.2449	19.4944	0.081	2.1103	0.0643	2.7533
13	92.7455	34.0183	3.8313	28.8865	3.0803	19.4944	0.009	3.6759	0.0732	4.4079
14	77.6634	51.1979	50	4.9573	3.0318	19.4944	0.002	4.2344	0.0812	5.0464
15	71.8947	16.5387	21.6223	34.1481	3.2188	19.4944	0.001	5.3441	0.0802	6.1461
16	106.8372	0	10.638	0	3.3141	19.4944	0.076	3.6672	0.0765	4.4322
17	68.1133	75	22.7282	3.0185	2.8464	19.4944	0.3	2.7675	0.0643	3.4105
18	74.0076	55.0592	19.5449	18.4296	2.3628	19.4944	0.049	2.3243	0.0723	3.0473
19	97.2919	50.794	4.859	11.5688	2.5268	19.4944	0.001	6.4421	0.0724	7.1661
20	71.5136	21.7212	0	5.03	2.7814	19.4944	0.211	5.5514	0.0756	6.3074
21	100.2235	9.6658	11.7449	45	2.7304	19.4944	0.077	3.7657	0.0722	4.4877
22	86.3776	24.4281	12.1505	3.4134	3.4719	19.4944	0.046	2.7771	0.0745	3.5221
23	78.5634	39.7367	23.2994	4.266	3.4058	19.4944	0.05	2.4587	0.0687	3.1457
24	78.5748	55.0442	38.8994	45	2.291	19.4944	0.001	2.2131	0.0564	2.7771
25	84.8562	2.6146	43.3555	9.9554	2.638	19.4944	0.016	2.4415	0.0764	3.2055
		**EAPL=**$\sum \Delta$**sc**$\cdot$**APL**					**3.4163**			
Case 16		**EAVD**=$\sum \Delta$**sc**$\cdot$**AVD**					**0.0699**			

### Module three

**Figure 6:**
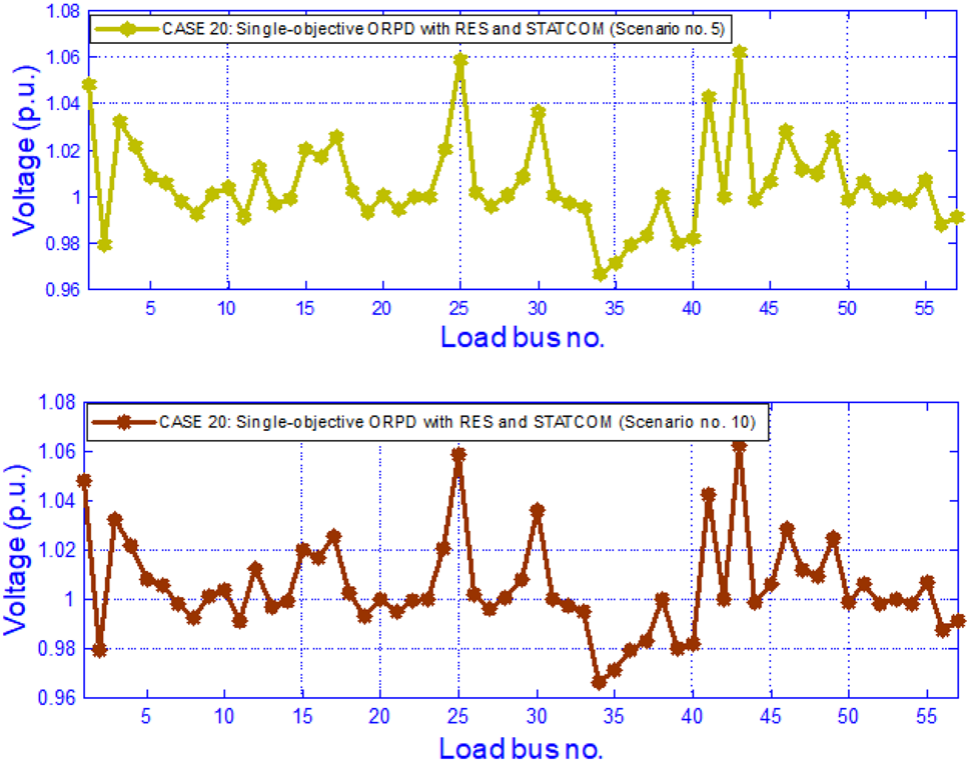
Load bus voltage deviations for case-20 at Scenario no 5 & Scenario no 10.

**Figure 7:**
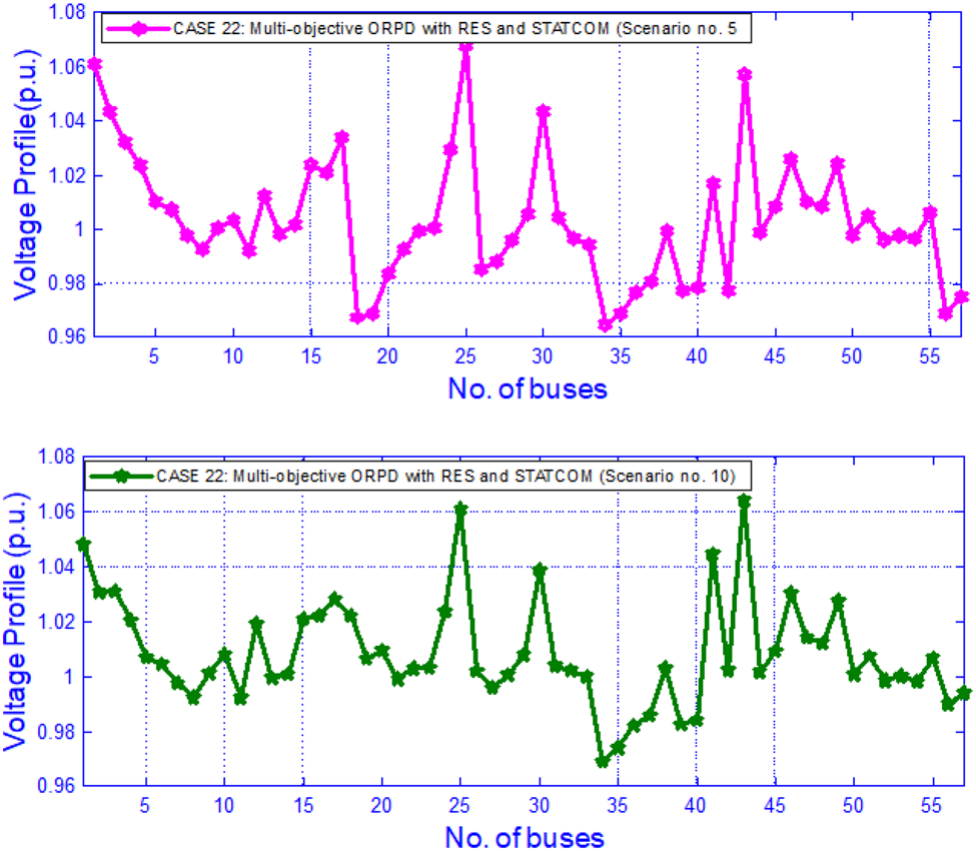
Load bus voltage deviations for case-22 at Scenario no 5 & Scenario no 10.

In this test module, the summary of the test system is given in Table 12 where IEEE 57 bus network is being considered. The scenarios, the % of loads, shearing of wind power, solar power and hydro power with scenario probabilities are furnished at Table 14. There are 6 cases (five single and one multi-objective) that have been taken care in this module from Case 17 to 22 and are mentioned earlier in Table 3. The outcomes of Case 17 to Case 21 (single objective) depending on scenarios are presented on Table 15 while the outcomes for Case 22 (multi-objective) are provided in Table 16. The variation of bus voltages for Case 20 under scenario 5 and 10 is given in Fig. 6. The relative hikes in EAPL, EAVD & EVSI in Case 22 is noticeable with respect to Case 17,20,21 respectively is due to the consideration of Multi objective in Case 22 while Case 17,20 &21 were single objective cases. Fig. 7 displays the fluctuation of voltages over buses for Case 22 on scenario 5 and 10.

From the overall simulation study, the superiority of DTBO to compute the efficacy of ORPD solutions has become starkly apparent over other recent optimization algorithms under all these cases, when the obtained test results are compared with the results which were presented in the literature on the same experimental platform. It is also evident from the simulation study that inclusion of STATCOM can significantly improve system’s performance. A brief overview of the IEEE 57-bus system are listed in Table 13.

**Table 12:** IEEE 57-bus details.

Items	Quantity	Details
Buses	Fifty-seven	^66^-^67^
Branches	Eighty	^66^^66^-^67^
Thermal units	Five	$B_1~\text{( }Swing),~B_2,~B_3 ~\text {and}~B_8$
WP unit	Two	$B_8~\text {and}~B_{12}$
Solar PV unit	One	$B_{9}$
HP unit	One	$B_{12}$
		$$L_{19},~~L_{20},~L_{31},~L_{35},~~L_{36},~L_{37} ~L_{41},~~L_{46},~L_{54},$$
Transformer	Seventeen	$L_{58},~~L_{59},~L_{65},L_{66},~~L_{71},~L_{73},~L_{76}, \text {and}~L_{80}$:
Control variables	Twenty-seven	$V_{G}$:Seven; $TR_{tran}$:Seventeen; $Q_c$:Three
Load demand	-	1250.8MW, 336.4MVAr
Range of load bus voltage	Fifty	0.95–1.05*p*.*u*.
STATCOM	One	-
Compensator	three	$QC_{18}$, $QC_{25}$ and $QC_{35}$

**Table 13:** An overview of IEEE 57- bus System.

	Bus no.	Pg (min)	Pg (max)	Qg (min)	Qg(max)	Setting of thermal unit (Case 17–22)
0.97	1	0	576	−140	200	Swing
0.97	2	30	100	−17	50	50
0.97	3	40	140	−10	60	60
0.97	6	30	100	−8	25	-
0.97	8	100	550	−140	200	400
0.97 Thermal	9	30	100	−3	9	-
Wind-6		0	75	−30	35	Variable
PV-9		0	50	−20	25	Variable
Wind+Hyd-12		100	410	−150	155	Variable

**Table 14:** Different loading of different sources for RESs based IEEE 57 bus.

Scenario no.	%Loading	Wind firm at bus 6 $P_{Wind1}$ (MW)	Solar at bus 9 $P_{Solar}$ (MW)	Wind firm and hydro plant at bus 12 $P_{Wind2}+P_{hydro}$ (MW)	Scenario probability $\Delta _{sc}$
1	85.4998	49.4094	22.7142	15.9291	0.005
2	93.0663	0	39.1718	22.1766	0.001
3	84.2514	34.5438	44.8937	15.9362	0.004
4	76.5664	3.7748	15.2782	33.1327	0.003
5	80.3714	9.544	14.4837	3.356	0.004
6	98.3753	56.9798	50	48.217	0.005
7	87.0929	75	49.3884	40.0138	0.001
8	88.9426	35.8258	3.3574	3.2402	0.04
9	92.3917	5.689	0	10.1301	0.011
10	110.5316	74.2512	4.8308	9.2394	0.005
11	81.5345	67.6788	37.7938	16.664	0.001
12	76.5164	27.4927	10.0666	19.4944	0.081
13	92.7455	34.0183	3.8313	31.9669	0.009
14	77.6634	51.1979	50	7.989	0.002
15	71.8947	16.5387	21.6223	37.3669	0.001
16	106.8372	0	10.638	3.3141	0.076
17	68.1133	75	22.7282	5.8649	0.3
18	74.0076	55.0592	19.5449	20.7923	0.049
19	97.2919	50.794	4.859	14.0956	0.001
20	71.5136	21.7212	0	7.8113	0.211
21	100.2235	9.6658	11.7449	47.7304	0.077
22	86.3776	24.4281	12.1505	6.8853	0.046
23	78.5634	39.7367	23.2994	7.6718	0.05
24	78.5748	55.0442	38.8994	47.291	0.001
25	84.8562	2.6146	43.3555	12.5934	0.016

**Table 15:** Results of Single-objective functions for IEEE 57 bus with RESs for ORPD.

Scenario no.	Scenario-based Ploss (MW) (Case-17)	Scenario-based Qloss (MVAr) (Case 18)	Scenario-based VD (p.u.) (Case 20)	Scenario-based VSI (p.u.) (Case 21)
1	7.870	−156.562	0.6876	0.1022
2	10.780	−145.662	0.6561	0.1102
3	9.334	−144.786	0.7112	0.1783
4	9.786	−103.563	0.6266	0.0998
5	8.897	−109.892	0.7861	0.1182
6	8.320	−110.632	0.6564	0.1128
7	10.673	−131.892	0.6998	0.1132
8	9.128	−145.672	0.6345	0.1354
9	10.688	−128.782	0.6453	0.1482
10	13.678	−174.686	0.6895	0.2073
11	7.675	−121.562	0.5342	0.1152
12	10.786	−109.588	0.6897	0.1382
13	11.778	−157.892	0.7108	0.1278
14	9.123	−99.893	0.6781	0.1134
15	8.987	−98.354	0.6786	0.1532
16	11.340	−168.671	0.6342	0.1982
17	7.998	−93.563	0.6196	0.0897
18	9.453	−121.672	0.7670	0.1372
19	11.675	−123.345	0.7786	0.1651
20	9.067	−97.356	0.7342	0.1280
21	14.908	−159.675	0.6908	0.1967
22	9.675	−112.345	0.7001	0.1500
23	9.778	−102.526	0.6897	0.1648
24	8.231	−105.571	0.7003	0.1176
25	11.785	−121.672	0.7520	0.1377
Case 17: **EAPL**=$\sum \Delta$**sc**$\cdot$**APL**			9.698	
Case 18: **EQL**=$\sum \Delta$**sc**$\cdot$**Qloss**			−113.9339	
Case19: **Cost of STATCOM**			27.68	
Case20: ** EAVD**= $\sum \Delta$**sc**$\cdot$**AVD**			0.6767	
Case21: ** EVSI**=$\sum \Delta$**sc**$\cdot$**VSI**			0.1322

**Table 16:** Multi-objective Results for IEEE 57 bus with RESs for ORPD.

Scenario no.	Scenario-based Ploss (MW)	Scenario-based VD (p.u.	Scenario-based VSI	Objective value $LVDVSI_{obj}$= $\lambda _l~P_{loss}$+$\lambda _{vd}VD$+$\lambda _{vsi}VSI$) (Case 22)
1	7.987	0.7234	0.1675	16.8960
2	10.884	0.7209	0.1897	19.9900
3	9.786	0.7562	0.2012	19.3600
4	9.991	0.6786	0.1530	18.3070
5	9.045	0.7998	0.1675	18.7180
6	8.552	0.7021	0.1342	16.9150
7	10.879	0.7231	0.1786	19.8960
8	9.564	0.6897	0.1657	18.1180
9	10.897	0.6904	0.1665	19.4660
10	13.897	0.7453	0.2342	23.6920
11	7.776	0.5786	0.1456	15.0180
12	10.987	0.7851	0.1554	20.3920
13	11.897	0.7786	0.1786	21.4690
14	9.564	0.7459	0.1342	18.3650
15	9.342	0.7231	0.1872	18.4450
16	11.766	0.6897	0.2101	20.7640
17	8.231	0.6786	0.1008	16.0250
18	9.678	0.7997	0.1421	19.0960
19	11.987	0.8002	0.1786	21.7750
20	9.387	0.7675	0.1445	18.5070
21	15.234	0.7342	0.1999	24.5750
22	9.779	0.7987	0.1567	19.3330
23	9.897	0.7231	0.1776	18.9040
24	8.453	0.7773	0.1439	17.6650
25	11.992	0.7897	0.1498	21.3870
		Case 22		
**EAPL**=$\sum \Delta$**sc**$\cdot$**APL=**			9.9633 MW	
** EAVD**= $\sum \Delta$**sc**$\cdot$**AVD=**			0.7299 p.u.	
** EVSI**=$\sum \Delta$**sc**$\cdot$**VSI=**			0.1461	
$\lambda _l=1$;	$\lambda _{vd}=10$;	$\lambda _{vsi}=10$		

### Module Four

In test Module four, IEEE 118 bus network has been chosen where RESs and STATCOM devices are also being added with the system. Here, a single wind farm is placed in bus 25, a PV unit is kept at bus 40, a combination of wind unit and hydro unit is connected to bus 70. The 25 scenarios, % loading, contribution of sole wind farm, PV unit, combined wind-hydro unit, and probabilities of scenarios considered in this module are referred to Table 14 which was also used in Module 3. As mentioned in Table 3, cases 23 to 25 (as single objective) & case 26 (as a multi-objective) are examined. The results obtained for Cases 23 to 25 & case 26 are placed in Table17 & 18 respectively.

**Table 17:** Single-objective ORPD evaluted cases with time-varying demand and uncertain renewable power with STATCOM (IEEE 118 Bus system).

Scenario no.	Scenario-based Ploss (MW) (Case-23)	Scenario-based VD (p.u.) (Case 24)	Scenario-based VSI (p.u.) (Case 25)
1	111.893	0.7665	0.1786
2	117.563	0.7877	0.1889
3	109.998	0.6988	0.1567
4	97.783	0.6791	0.1556
5	108.983	0.7281	0.1763
6	121.903	0.7882	0.1787
7	110.673	0.7483	0.1733
8	112.783	0.7553	0.1711
9	119.893	0.7711	0.1798
10	131.783	0.8001	0.1801
11	108.783	0.6899	0.1698
12	98.807	0.6798	0.1678
13	112.881	0.7423	0.1745
14	97.339	0.7167	0.1722
15	87.709	0.6987	0.1687
16	129.897	0.7987	0.1799
17	78.909	0.6676	0.1676
18	88.897	0.7124	0.1704
19	121.786	0.7865	0.1779
20	89.897	0.7453	0.1755
21	124.901	0.7986	0.1801
22	89.995	0.7112	0.1703
23	83.449	0.6987	0.1689
24	84.986	0.6999	0.1701
25	98.903	0.7568	0.1766
Case 23: **EAPL**=$\sum \Delta$**sc**$\cdot$**APL**			95.0539
Case24: ** EAVD**= $\sum \Delta$**sc**$\cdot$**AVD**			0.7202
Case25: ** EVSI**=$\sum \Delta$**sc**$\cdot$**VSI**			0.1722

**Table 18:** Multi-objective ORPD evaluted cases with time-varying demand and uncertain renewable power with STATCOM (IEEE 118 bus system).

Scenario no.	Scenario-based Ploss (MW)	Scenario-based VD (p.u.	Scenario-based VSI	Objective value $LVDVSI_{obj}$= $\lambda _l~P_{loss}$+$\lambda _{vd}VD$+$\lambda _{vsi}VSI$) (Case 26)
1	112.563	0.7723	0.1799	122.0852
2	118.452	0.7998	0.1901	128.3511
3	110.563	0.7003	0.1634	119.2002
4	98.674	0.6896	0.1567	107.1367
5	110.563	0.7345	0.1775	119.6832
6	123.003	0.7998	0.1799	132.7997
7	111.783	0.7562	0.1776	121.1212
8	113.674	0.7667	0.1756	123.0973
9	120.453	0.7873	0.1811	130.1372
10	132.784	0.8113	0.1845	142.7423
11	109.897	0.6998	0.1723	118.6181
12	99.663	0.6887	0.1698	108.2477
13	113.983	0.7564	0.1765	123.3122
14	98.672	0.7206	0.1788	107.6664
15	88.672	0.7012	0.1699	97.3833
16	130.785	0.8011	0.1823	140.6185
17	79.537	0.6782	0.1742	88.0606
18	89.453	0.7223	0.1778	98.4536
19	123.009	0.7889	0.1823	132.7212
20	90.675	0.7499	0.1811	99.9854
21	125.563	0.8012	0.1878	135.4533
22	90.783	0.7167	0.1781	99.7312
23	84.784	0.7012	0.1703	93.4993
24	86.112	0.7011	0.1745	94.8683
25	100.673	0.7671	0.1802	110.1462
		Case 26		
**EAPL**=$\sum \Delta$**sc**$\cdot$**APL=**			95.8387MW	
** EAVD**= $\sum \Delta$**sc**$\cdot$**AVD=**			0.7275 p.u.	
** EVSI**=$\sum \Delta$**sc**$\cdot$**VSI=**			0.1774	
$\lambda _l=1$;	$\lambda _{vd}=10$;	$\lambda _{vsi}=10$		

### Module Five

**Table 19:** Single-objective ORPD evaluated cases with time-varying demand and uncertain renewable power with STATCOM (IEEE 300 Bus system).

Scenario no.	Scenario-based Ploss (MW) (Case-27)	Scenario-based VD (p.u.) (Case 28)	Scenario-based VSI (p.u.) (Case 29)
1	371.563	3.1120	0.3217
2	378.786	3.1672	0.3356
3	370.897	3.1101	0.3167
4	368.987	3.0789	0.3080
5	369.801	3.1008	0.3164
6	376.811	3.1563	0.3267
7	374.782	3.1652	0.3211
8	375.003	3.2011	0.3260
9	379.093	3.2041	0.3267
10	401.861	3.3755	0.3323
11	369.998	3.1372	0.3110
12	365.902	3.1156	0.3120
13	373.995	3.1783	0.3201
14	368.908	3.0134	0.3001
15	364.894	3.0111	0.3000
16	396.892	3.2001	0.3211
17	356.779	3.0001	0.2980
18	367.911	3.1025	0.3178
19	378.774	3.2190	0.3267
20	368.662	3.1178	0.3130
21	384.673	3.2152	0.3221
22	377.822	3.1982	0.3197
23	370.554	3.1452	0.3145
24	371.452	3.1787	0.3187
25	378.338	3.2014	0.3210
Case 27: **EAPL**=$\sum \Delta$**sc**$\cdot$**APL**			369.5651
Case28: ** EAVD**= $\sum \Delta$**sc**$\cdot$**AVD**			3.1077(p.u.)
Case29: ** EVSI**=$\sum \Delta$**sc**$\cdot$**VSI**			0.3114

**Table 20:** Multi-objective ORPD evaluted cases with time-varying demand and uncertain renewable power with STATCOM (IEEE 300 bus system).

Scenario no.	Scenario-based Ploss (MW)	Scenario-based VD (p.u.	Scenario-based VSI	Objective value $LVDVSI_{obj}$= $\lambda _l~P_{loss}$+$\lambda _{vd}VD$+$\lambda _{vsi}VSI$) (Case 30)
1	372.675	3.1564	0.3277	407.5164
2	379.334	3.1786	0.3453	414.5733
3	371.564	3.1678	0.3231	406.4733
4	369.674	3.1298	0.3112	404.0843
5	370.779	3.1457	0.3231	405.4666
6	378.786	3.1667	0.3334	413.7873
7	376.010	3.1908	0.3342	411.2599
8	377.097	3.2345	0.3387	412.8294
9	380.564	3.2334	0.3897	416.7953
10	404.899	3.3897	0.3564	442.3597
11	371.102	3.1564	0.3445	406.1111
12	366.112	3.1334	0.3235	400.6813
13	374.892	3.2003	0.3334	410.2293
14	369.646	3.0567	0.3453	403.6662
15	366.546	3.0492	0.3334	400.3716
16	397.453	3.2123	0.3452	433.0282
17	358.673	3.1034	0.3109	392.8162
18	368.563	3.1675	0.3278	403.5163
19	380.324	3.2786	0.3345	416.4554
20	370.435	3.1556	0.3298	405.2892
21	385.908	3.2786	0.3445	422.1391
22	379.664	3.2109	0.3234	415.0073
23	371.675	3.1887	0.3221	406.7833
24	371.999	3.1998	0.3289	407.2856
25	379.710	3.2342	0.3338	415.3899
		Case 30		
**EAPL**=$\sum \Delta$**sc**$\cdot$**APL=**			371.0125MW	
** EAVD**= $\sum \Delta$**sc**$\cdot$**AVD=**			3.1635p.u.	
** EVSI**=$\sum \Delta$**sc**$\cdot$**VSI=**			0.3264	
$\lambda _l=1$;	$\lambda _{vd}=10$;	$\lambda _{vsi}=10$		

The IEEE 300 bus system has been selected for test Module 5, and the system will also include RESs and STATCOM devices. In this instance, bus 84 has a single wind farm, bus 108 carries a photovoltaic unit, and bus 152 keeps a wind- hydro unit combo. Referring to Table 14, which was also utilized in the previous two modules, are the 25 scenarios, % loading, contribution of a single wind farm, PV unit, combined wind-hydro unit, and probability of scenarios taken into consideration in this module. Cases 27 to 29 (as single objective) and case 30 (as a multi-objective) are analyzed here, as indicated in Table 3. Table19 and Table20 include the results for Cases 27 to 29 and Case 30 respectively.

## Conclusions

Using DTBO across five test setups, as demonstrated in five study modules, the ORPD problem has been tackled in the current work. The first one looks at a typical IEEE 30-bus network, and the second one looks at a traditional network that has been reconfigured with RESs connected. In the third, forth & the fifth modules of the study respectively IEEE 57 bus, 118 bus & 300 bus network have been used as test setup. A deterministic environment is used for the study’s earlier phases, and in the latter sections, the approach of scenario development and reduction procedure is used to address the stochasticity of load demand and RESs. Scenarios are created using MCS, and they are then condensed into a manageable number utilizing BRA. In this regard, appropriate PDFs of load demand and RESs are also being taken into account. The study has two objectives: first, it aims to minimize APL, AVD, and VSI individually as a single target; second, it aims to minimize APL and AVD jointly as a multi-objective. Experiments are run in both test configurations, once with STATCOM taken into account and once without. The results of the experiments show that DTBO is more effective than modern optimization algorithms in both deterministic experimental setups and test scenarios where volatility is prevalent. Additionally, it has been verified that all network constraints are currently kept within predetermined bounds. One intriguing result of this work is that, with regard to the ORPD issue, using STATCOM is highly beneficial due to the decrease in the system’s APL, AVD, and VSI. STATCOM continues to be incredibly effective in both fixed and uncertain loading scenarios. Despite the presence of unpredictable RESs in the power network, STATCOM nevertheless improves system performance.Further experiments can be conducted using higher ordered IEEE standard networks.

From simulation results it has been observed that for IEEE 30 bus, the average power loss (APL) is 4.5086 MW. However, after utilizing STATCOM, the APL is reduced by 5.3% and with the integration f renewable sources, the APL is reduced by 41%, and for both STATCOM and RESs system, it decreases to 43.6%. Hence, STATCOM and RES help to reduce the power losses using DTBO approach. Furthermore, the average voltage deviation (AVD) is improved by 97.4 % with incorporating STATCOM-RESs. Voltage stability index (VSI) is improved by 26.9% with scheduling STATCOM and renewable sources (RESs). For the multi-objective situation, APL & AVD both are simultaneously improved to 5.0701(MW) & 0.1221 (*p*.*u*.), respectively, after incorporating STATCOM and RESs using DTBO. Voltage deviation converges at 40 iterations for simulation study having STATCOM but for without STATCOM, it takes 80 iterations to converge. Similarly for voltage stability index with STATCOM converge 4 iterations earlier as compared to that of without STATCOM system. Again, for large scale IEEE 57 bus system, The DTBO approach incorporating STATCOM and RESs provides optimal results. So, for both IEEE 30 and IEEE 57 bus systems, DTBO proof its superiority and robustness satisfactorily. Furthermore, the study also covers the experiments on IEEE 118 & 300 bus network in Module four & five respectively where the outcomes are also remarkably well.

## Supplementary Information

Supplementary Information.

## Data Availability

The authors confirm that the data supporting the findings of this study are available on request to Dr. Tushnik Sarkar (tushnik.sarkar@bcrec.ac.in).
