# Supplementary material for: Optimal allocation of STATCOM for multi-objective ORPD problem on thermal wind solar hydro scheduling using driving training based optimization
Source: Sci Rep. 2025 Jun 4;15:19594. doi: 10.1038/s41598-025-02636-1 (PMC12137891; doi:10.1038/s41598-025-02636-1)
Supplement: Supplementary file 1 — Supplementary Information. [file 41598_2025_2636_MOESM1_ESM.pdf]

Appendix

Operating limits of active and reactive power of IEEE 30-bus system are illustrated in Table A1

Table A1

Appendix:Generator data for IEEE 30-bus system

Bus no.	Pg (min)	Pg (max)	Qg (min)	Qg (max)	Setting of thermal unit (Case 1-8)	Setting of thermal unit (Case 9-16)
1	50	200	-20	150	Swing	Swing
2	20	80	-20	60	75	75
5 (thermal)	15	50	-15	62.5	40	–
5(wind I)	0	75	-30	35		Variable
8	10	35	-15	48.7	30	30
11(thermal)	10	30	-10	40	25	–
11(PV)	0	50	-20	25		Variable
13(thermal)	12	40	-15	44.7	30	–
13(wind II)	0	45	-20	35		Variable
13 (Hydro)	0	5	-10	20		Variable
